# Ligand-Bound Forced Degradation as a Strategy to Generate Functionally Relevant Analytical Challenge Materials for Assessment of CQAs

**DOI:** 10.3389/fmolb.2022.789973

**Published:** 2022-04-11

**Authors:** John P. Giddens, John E. Schiel

**Affiliations:** ^1^ National Institute of Standards and Technology, Institute for Bioscience and Biotechnology Research, Rockville, MD, United States

**Keywords:** methionine oxidation, NISTmAb, mass spectrometry, surface plasmon resonance, analytical materials

## Abstract

Therapeutic monoclonal antibodies (mAbs) contain a variety of amino acids that are susceptible to enzymatic, chemical, and physical modifications. These modifications can happen throughout production, purification, formulation, and storage and many are known to affect the biological activity of a mAb. Methods that are able to characterize and evaluate these attributes are critical in order to understand how they might alter biological activity. Methods capable of site-specific monitoring of these critical quality attributes are extremely valuable to biopharmaceutical research but also require well-defined materials with site-specific attribute modifications. Here, we describe the development and application of a strategy to generate functionally relevant analytical challenge materials that have unique site-specific attributes. This method involves the use of a ligand that is bound to the mAb during oxidative stress resulting in unique oxidation patterns with some methionine residues protected while others are exposed to oxidation. These unique materials were used to develop a rapid surface plasmon resonance (SPR) assay that could detect methionine oxidation in both the Fab and Fc regions using specific molecular probes. The addition of uniquely oxidized materials to our data set enabled us to determine specific methionine residues vital to binding. Further analysis showed that antibody oxidation could also be rapidly detected in multiple domains from qualitative thermal melting using intrinsic tryptophan fluorescence. Methionine oxidation of an antibody was explored in this study, but we envision this method could be useful to explore structure function relationships of a variety of antibody modifications and modifications to other biologically relevant protein drugs.

## Introduction

Therapeutic monoclonal antibodies (mAbs) are an important class of biological therapeutics widely used for the treatment of cancer, autoimmune diseases, and various infectious diseases. mAbs are susceptible to a variety of enzymatic, chemical, and physical modifications throughout production, purification, formulation, and storage that are known to alter their biological properties. The result is a complicated mixture of product variants that can pose a challenge to current analytical measurements and complicates interpretation of a modification’s impact on stability and/or function. The potential impact of product variants are evaluated during mAb development by imparting intentional stressors in excess to induce artificial changes in product quality, a process called forced degradation. Later in development, stability testing is performed on the formulated drug substance or drug product, wherein stressors model more real-world intrusions (Li et al., 2015).

Post-translational modifications (PTMs) are changes in the polypeptide occurring after translation due to enzymatic processing, although chemically induced and/or exacerbated changes are typically also included in a broader definition (Li et al., 2015). One of the most common and pervasive PTMs that affects mAbs is oxidation, which can be caused by peroxides, metal ions, and light. Methionine is the amino acid most susceptible to oxidation in mAbs, while oxidation of several other amino acids like tryptophan, cysteine, lysine, and histidine have also been reported (Chen et al., 2019). Methionine oxidation in the Fc region has been widely studied and shown to cause reduced Fc mediated activity by decreasing interactions with the neonatal Fc receptor (FcRn) and other Fc receptors (Bertolotti-Ciarlet et al., 2009; Pan et al., 2009; Gao et al., 2015; Cymer et al., 2017). Specifically, oxidation of Met 255 and Met 431 (actual sequence number varies owing to differing complementarity-determining region (CDR) lengths and often is referred to as Met 252 and Met 428) have been demonstrated to cause a decrease in binding of FcRn. Oxidation of amino acids in the CDR has also been implicated in decreased antigen binding (Dashivets et al., 2016). Others have shown that Met oxidation can interfere with immunoglobulin G (IgG) oligomerization, which is needed for C1q binding and complement dependent cytotoxicity (Mo et al., 2016). Investigation of the biological impact of antibody methionine oxidation is important for many aspects of antibody function, and higher resolution information on the effects of a specific oxidized methionines are of great interest.

Because of the global nature of stress protocols, all susceptible methionine residues are oxidized, albeit at varying rates. The rate of oxidation depends on a variety of factors, with solvent accessible surface area being the major contributing factor (Pan et al., 2009; Sokolowska et al., 2017). Although many studies have demonstrated correlations between the biological impact of IgG and methionine oxidation, direct assessment of a specific methionine residue can be difficult to evaluate because it is challenging to generate materials with oxidation at a specific methionine residue. Genetic manipulation of specific methionine residues has been used; methionine to leucine to block oxidation at a site or methionine to glutamine to mimic an oxidized methionine. These genetic modifications allowed for the expression of mAbs with site selective oxidation mimics useful in deciphering structure-function relationships of IgG binding to FcRn (Gao et al., 2015). However these sequence-engineered materials have altered primary amino acid sequence and do not directly represent the same product. Another approach attempted to enrich Met-oxidized species using affinity chromatography with ligands that can distinguish methionine oxidation at certain residues (Stracke et al., 2014). This approach can enrich a certain population of Met-oxidized antibodies, but is rather cost prohibitive at scale and still cannot provide sufficiently pure material.

As an alternative, we envisioned a method that allows for a binding partner known to interact with specific methionine residues to mask oxidation at that site when chemical oxidation was performed in the presence of that ligand. By decreasing the solvent exposure, the kinetic rate of oxidation should decrease drastically. The publicly available IgG1κ monoclonal antibody Reference Material 8671, NISTmAb, was chosen as an example material due to its usefulness in evaluation and development of emerging analytical measurement technologies (Schiel et al., 2015). Here we present a method to generate functionally relevant, selectively oxidized materials that are useful as analytical challenge materials. We chose to generate a library of these selectively oxidized mAbs in combination with traditionally oxidized samples and characterized each sample with mass spectrometry to access the quantity of residue-specific modifications. Furthermore, we use these unique challenge materials to demonstrate how a rapid surface plasmon resonance-based assay and a thermal unfolding assay can differentiate the impact of oxidation at different regions of the mAb, providing highly valuable information that could be useful in the assessment of critical quality attributes of antibody therapeutics.

## Materials and Methods

### Preparation of the Stressed Samples

#### NISTmAb Alone vs. NISTmAb With Protein A in Solution

Two sets of oxidized samples were generated, one with NISTmAb alone (Nox 1, Nox 3, and Nox 6) and the other with a mixture of NISTmAb and protein A (PAox 1, PAox 3, PAox 6). Nox samples were generated as follows: 100 µL of NISTmAb at 10 mg/ml was added to each vial followed by the addition of 22.6 µL of phosphate-buffered saline (137 mmol/L NaCl, 2.7 mmol/L KCl, 10 mmol/L Na_2_HPO_4_) (PBS) pH 7.4 and 4.2 µL of 30% (w/w) H_2_O_2_ to give a final concentration of H_2_O_2_ of 1% and a final concentration of NISTmAb of 7.88 mg/ml. Samples were incubated at 25°C, protected from light. Samples were centrifuged for 5 min at 10,000 rpm and reactions were stopped by buffer exchanging back into formulation buffer (pH 6.0, 25 mmol/L L-Histidine) using zeba spin desalting columns 7K MWCO (Thermo Scientific) at the following time points: 1, 3, and 6 h to generate ^1%^Nox_1hr_, ^1%^Nox_3hr_, and ^1%^Nox_6hr_. PAox samples were generated as follows: 100 µL of NISTmAb at 10 mg/ml was added to each vial followed by the addition of 22.6 µL of protein A at 25 mg/ml in PBS and 4.2 µL of 30% (w/w) H_2_O_2_ to give a final concentration of H_2_O_2_ of 1% and a final concentration of NISTmAb of 7.88 mg/ml. Samples were incubated at 25°C, protected from light. Samples were centrifuged for 5 min at 10,000 rpm and reactions were stopped by buffer exchanging back into formulation buffer (pH 6.0, 25 mmol/L L-Histidine) using zeba spin desalting columns 7K MWCO (Thermo Scientific) at the following time points: 1, 3, and 6 h to generate ^1%^PAox_1hr_, ^1%^PAox_3hr_, and ^1%^PAox_6hr_.

#### Standard in Solution Oxidation Time Course

To achieve forced oxidation, 8.01 µL of 30% (w/w) H_2_O_2_ was added directly to a 800 µL vial (8 mg at 10 mg/ml) of NISTmAb RM 8671 samples to obtain a final concentration of 0.3% H_2_O_2_ and 9.9 mg/ml of NISTmAb. Samples were incubated at 25°C, protected from light. Samples were centrifuged for 5 min at 10,000 rpm and reactions were stopped by buffer exchanging back into formulation buffer (pH 6.0, 25 mmol/L L-Histidine) using zeba spin desalting columns 7K MWCO (Thermo Scientific) at the following time points: 30 min, 1, 2, 4, 6, 8, 16, 32 h. A NISTmAb Bex sample was generated by buffer exchanging a NISTmAb RM 8671 vial into formulation buffer using the same method as oxidized samples. A NISTmAb control sample was also generated by incubating a NISTmAb vial without the addition of H_2_O_2_, at 25°C and protected from light for 32 h and then buffer exchanged into formulation buffer using the same method as oxidized samples. All buffer exchanged samples were measured for a concentration (all samples were between 7 mg/ml to 8 mg/ml) using a NanoDrop 1000C, aliquoted to 50 μL, and stored at −80°C.

#### Oxidation While Bound to Protein A Column

NISTmAb (8 mg) was loaded onto a 1 ml MabSelect SuRe protein A column (GE Lifesciences) and the column was washed with PBS pH 7.4 for 5 column volumes. To achieve forced oxidation a solution of H_2_O_2_ in PBS was run over the column at a flow rate of 0.5 ml/min for a specific amount of time. Five CPA samples were generated using a different amount of H_2_O_2_ and different exposure times: ^0.3%^CPA_2hr_, ^0.3%^CPA_4hr_, ^0.3%^CPA_6hr_, ^0.0375%^CPA_16hr_, and ^3%^CPA_1hr_ (Where the superscript denoted H_2_O_2_% and subscript denotes time exposed to H_2_O_2_ on the protein A column). After the oxidation, the column was washed with PBS pH 7.4 for 5 column volumes to get rid of any excess H_2_O_2_. Bound IgG was eluted using a citric acid solution (100 mmol/L pH 3.0) and quickly neutralized with 1.5 mol/L tris buffer pH 8.8. Fractions containing eluted NISTmAb were combined and concentrated using amicon ultra centrifugal filter unit 10 KDa (Millipore). Samples were then centrifuged for 5 min at 10,000 rpm and buffered exchanged back into formulation buffer (pH 6.0, 25 mmol/L L-Histidine) using zebra spin desalting columns 7K MWCO (Thermo Scientific) and aliquots were stored at −80°C. The concentrations of all samples were measured using a Nanodrop 2000 C system and ranged from 7 mg/ml to 8 mg/ml.

### LC-MS Analysis

#### IdeS Subunit Analysis

The antibody samples were diluted to 3 mg/ml into 0.25 mol/L Tris-HCl, pH 7.5 and one unit of IdeS protease was added for every 1 µg of antibody. The samples were digested by incubating the solution at 37°C for 30 min. The digested samples were then diluted 1:10 into a denaturing buffer comprised of 6 mol/L guanidine HCl in 0.25 mol/L Tris-HCl, pH 7.5. The mAbs were then reduced by adding dithiothreitol (DTT) to a final concentration of 25 mmol/L for 60 min at 45°C. The samples were then buffer exchanged into 0.1% formic acid (FA) and 10% acetonitrile in LC-MS grade water using a zebra spin filter column. 2.5 µg of each peptide digest was injected onto a Waters UPLC Protein Ethylene Bridged Hybrid C4 column (150 × 2.1 mm i.d. 1.7 μm BEH particles, 300 Å) set to 60°C and analyzed by liquid chromatography-electrospray ionization-mass spectrometry (LC-ESI-MS/MS) using an Agilent 1200 Infinity II series LC system coupled to an Agilent 6545XT AdvanceBio LC/Q-TOF. The chromatographic method was initiated with 80% Mobile Phase A (0.1% FA in water) and 20% Mobile Phase B (0.1% FA in acetonitrile) with a flow rate of 0.4 ml/min. The separation was achieved over 20 min starting with a 5 min isocractic hold at 20% B followed by a gradient to 45% B in 15 min. The column was then washed by ramping up to 95% B in 1 min followed by a 4 min hold at 95% B. The column was equilibrated by returning the flow to 20% B followed by a 2 min hold at 20% B. The MS instrument was operating in positive ion mode with the following source settings: gas temp 350°C, drying gas 8 L/min, nebulizer 2.4 × 10^5^ Pa, sheath gas temp 275°C, sheath gas flow 11 L/min, VCap 5000 V, nozzle voltage 1000 V, fragmentor 250 V, skimmer 65 V, Oct 1 RF Vpp 750 V. The mass range for MS1 was 300–3,200 m/z and data was acquired at 1 spectra/s.

#### Peptide Mapping Analysis

The antibody samples were prepared and digested following a previous publication (Mouchahoir and Schiel, 2018). Briefly, the samples were denatured in a buffer comprised of 6 mol/L guanidine HCl, 1 mmol/L ethylenediaminetetraacetic acid (EDTA) in 0.1 mol/L Tris-HCl, pH 7.8. The mAbs were then reduced with a final concentration of 5 mmol/L dithiothreitol (DTT) for 60 min at 4°C and alkylated with a final concentration of 10 mmol/L of iodoacetamide (IAM) for 60 min at 4°C. The samples were then buffer exchanged into 1 mol/L urea in 0.1 mol/L Tris, pH 7.8 and trypsin was added at a 1:18 (enzyme: sample) mass ratio. The digestion was incubated for 4 h at room temperature. The reaction was stopped by adding 0.1% formic acid (FA) in LC-MS grade water was added at a 1:1 volume ratio and the digests were stored at -80°C until analysis. 2.5 µg of each peptide digest was injected onto a Agilent Zorbax RRHD StableBond C18 column (150 mm × 2.1 mm i.d. 1.8 μm BEH particles, 300 Å) set to 40°C and analyzed by LC-ESI-MS/MS using an Agilent 1200 Infinity II series LC system coupled to an Agilent 6545XT AdvanceBio LC/Q-Tof. The chromatographic method was initiated with 99% Mobile Phase A (0.1% FA in water) and 1% Mobile Phase B (0.1% FA in acetonitrile) with a flow rate of 0.25 ml/min. The separation was achieved over 72 min starting with a 5 min isocractic hold at 1% B followed by a steep gradient to 10% B in 1 min ending with a gradient to 35% B in 64 min. The column was then washed by ramping up to 90% B in 2 min followed by a 5 min hold at 90% B and then back down to 1% B in 2 min followed by an isocratic at 1% B for 2 min. The gradient was then raised to 10% B over 2.5 min, then to 45% B in 8 min, and lastly to 90% B in 1.5 min. A final isocratic hold at 90% for 6 min was performed and the column was equilibrated by returning to 1% B for 14 min before the next sample. The MS instrument was operating in positive ion mode with the following source settings: gas temp 325°C, drying gas 13 L/min, nebulizer 2.4 × 10^5^ Pa, sheath gas temp 275°C, sheath gas flow 12 L/min, VCap 4000 V, nozzle voltage 500 V, fragmentor 175 V, skimmer 65 V, Oct 1 RF Vpp 750 V. The mass range for MS1 was 100 m/z to 2,400 m/z and 50 m/z to 2,400 m/z in MS2. Ions were selected for MS/MS with a narrow 1.3 m/z window then fragmented by collision induced dissociation using the formula, collision energy = 3.6*(m/z)/100 + 4.8. The MS2 conditions were as follows: Top 10, 3,000 counts abs threshold/0.001% rel threshold, active exclusion enabled with exclusion after 3 spectra and released after 0.2 min. The MS data was analyzed using Genedata Expressionist software. Briefly, raw data was imported into Genedata Expressionist and a workflow was run that includes background subtraction followed by retention time alignment, MS peak detection, charge assignment, MS/MS consolidation and peak detection. The MS1 mass tolerance was set at 10 ppm and MS2 mass tolerance was 50 ppm for peptide identification. The oxidation percentage of each Met residue was calculated by dividing the peak area of the oxidized peptide by the sum of the peak areas of both oxidized and nonoxidized peptide.

### Size Exclusion Chromatography Analysis

SEC analysis was performed according to a previously developed method (Turner et al., 2018). Briefly, all samples were analyzed on an Agilent high pressure liquid chromatography system using isocratic elution (100 mmol/L sodium phosphate supplemented with 250 mmol/L sodium chloride, pH 6.8) at 0.30 ml/min and monitored at 280 nm. 60 µg of antibody sample was injected onto a Waters Acquity UPLC Protein BEH SEC column (1.7 μm particle size, 200 Å pore size, 4.6 × 150 mm length).

### SPR Analysis

SPR experiments were performed using a Biacore T200 system (GE Healthcare) with analysis temperature set to 25°C and sample compartment temperature set to 15°C. Series S Sensor Chip CAP, PBS-P + Buffer 10x [0.2 mol/L phosphate buffer with 27 mmol/L KCl, 1.37 mol/L NaCl and 0.5% v/v Surfactant P20 (Tween 20)], and Biotin CAPture Kit were all obtained from GE Healthcare. A peptidic epitope of the NISTmAb with the sequence NSELLSLINDMPITNDQKKLMSNN and N-terminal acetylation, C-terminal amidation, and a C-terminal biotinylated lysine residue was synthesized by Genscipt. Recombinant biotinylated protein A (29989) and protein L (21189) were purchased from Thermo Fisher.

Measurements were conducted using a double-capture method *via* the oligonucleotide-immobilized CAP sensor chip, Biotin CAPture reagent (streptavidin bound to an oligonucleotide complementary to the strand on the CAP chip), and the various biotinylated ligands (protein A, F peptide, and protein L). Biotin capture reagent was injected for 300 s at a flow rate of 2 μL/min to capture approximately 3,000 response units (RU) followed by a 60 s injection at 5 μL/min into Fc2 of biotinylated protein A (0.02 μg/μL) to give a capture level of 275 RU to 305 RU, another 60 s injection at 5 μL/min into Fc3 of biotinylated F peptide (5 μg/μL) to give a capture level of 295 RU to 305 RU, and a final 60 s injection at 5 μL/min into Fc4 of biotinylated protein L (0.02 μg/μL) to give a capture level of 220 RU to 230 RU. Samples were diluted in running buffer (PBS-P+ pH 7.4) to a concentration of 200 nmol/L and flowed over each flow channel at a flow rate of 50 μL/min. Each complex was allowed to associate and dissociate for 100 and 300 s, respectively. Following the association and dissociation phases of the experiment, the chip was regenerated with an injection of regeneration buffer 1 for 120 s (6 mol/L guanidine-HCL, 0.25 mol/L NaOH) and injection of regeneration buffer 2 for 120 s (30% acetonitrile in 0.25 mol/L NaOH).

A binding affinity response point was taken at the maximum binding level at the end of the association phase for each sample. The RU value was normalized for each ligand by dividing each data point by the maximum value of NISTmAb Bex for that ligand in each experiment. The relative binding level for each sample to each ligand was calculated by averaging sample replicates over three independent experiments and the standard deviation along with %CV were also calculated.

### Thermal Unfolding Analysis

Thermal unfolding experiments were performed using a Tycho NT 6.0 system (NanoTemper). All samples were diluted to 1 mg/ml in formulation buffer (pH 6.0, 25 mmol/L L-Histidine) before analysis. Samples were heated from 35°C to 95°C over 3 min, and intrinsic fluorescence at 350 and 300 nm were monitored, providing a relative thermal stability for each sample. Unfolding profiles were generated by plotting the fluorescence ratio of 350 nm/330 nm. NanoTemper software then calculated the inflection temperatures by taking the first derivative of this ratio where max and min peaks correlate to inflection temperatures. The average value and standard deviation of inflection temperatures and initial ratio was calculated using three sample replicates.

### Statistical Analysis

Graphpad Prism version 9.1.2 was used for all statistical analysis. The unpaired *t*-test was performed with the following settings: Assume Gaussian distribution and two-tailed *p* value calculation. The nonlinear regression analysis was performed using either a straight line model for linear data or One phase decay model for nonlinear data using standard settings. The standard error of regression (Sy.x) was calculated and the 90% prediction bands (the area that 90% of future data points are expected) were also plotted. Replicates were accounted for by using the number of samples (N) and the standard deviation.

## Results and Discussion

### Method to Change Methionine Oxidation Kinetics at a Specific Site: Solution Phase Protein A Protection Proof of Principle

Oxidative stress of NISTmAb in the presence of protein A in solution was used to initially assess the ability of protein A to mask, and thereby protect, Fc methionine residues from oxidation. The levels of oxidation of NISTmAb alone and NISTmAb with protein A in solution (1:2 molar ratio) were compared after exposure to a strong accelerated oxidative stress condition, 1% hydrogen peroxide (H_2_O_2_) solution, for 1, 3, and 6 h. The global oxidation levels of both sets of oxidized samples, NISTmAb alone (^1%^Nox_1hr,_^1%^Nox_3hr,_^1%^Nox_6hr_) and NISTmAb with protein A (^1%^PAox_1hr_, ^1%^PAox_3hr_, ^1%^PAox_6hr_) were monitored by liquid chromatography mass spectrometry (LC-MS) and compared to the unstressed NISTmAb RM 8671. A rapid subunit mass analysis was employed which entailed enzymatic digestion with IdeS to specifically cleave IgG in the hinge region, resulting in three subunits after reduction of disulfide bonds: Fc/2, Fd’, and LC. A representative spectrum of all three subunits of NISTmAb RM 8671 is shown in Supplementary Figure S1. The analytical method was capable of identifying all previously reported proteoforms of the NISTmAb and deemed suitable for preliminary oxidation screening. A full list of identified masses, including observed and theoretical masses for all proteoforms, can be found in Supplementary Table S1.

**FIGURE 1:**
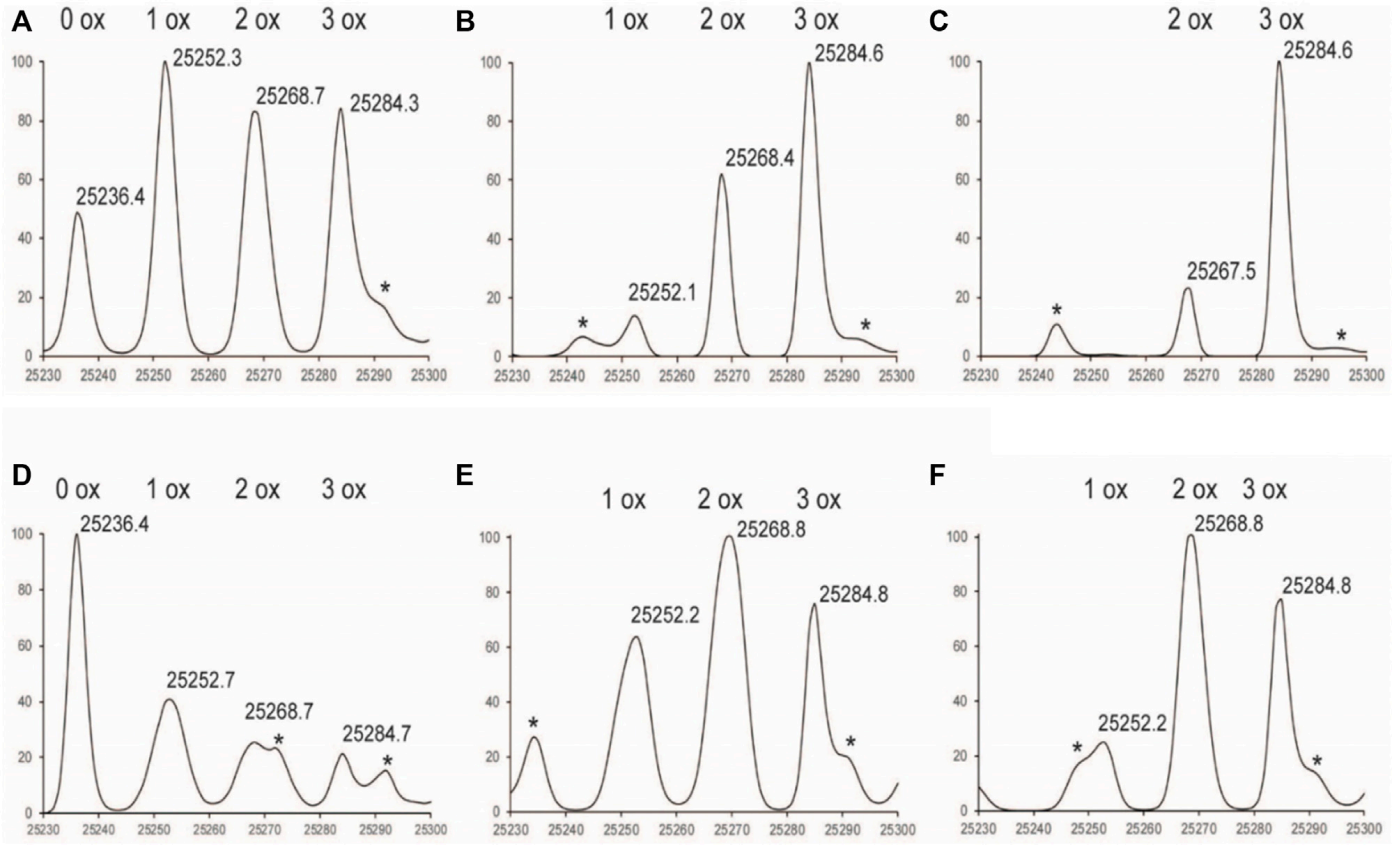
Deconvoluted LC-MS Spectrum of Fc subunit zoomed in to G0F **(A)** ^1%^Nox_1hr_ **(B)** ^1%^Nox_3hr_ **(C)** ^1^^%^Nox_6hr_ **(D)** ^1%^PAox_1hr_ **(E)** ^1%^PAox_3hr_ **(F)** ^1%^PAox_6hr_ *Denotes adduct peaks that do not correspond to actual oxidized antibody peaks.

**FIGURE 2:**
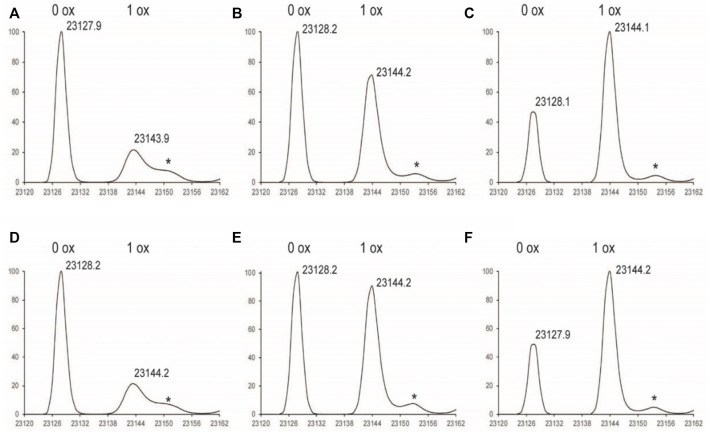
Deconvoluted LC-MS Spectrum of LC subunit **(A)** ^1%^Nox_1hr_ **(B)** ^1%^Nox_3hr_ **(C)** ^1%^Nox_6hr_ **(D)** ^1%^PAox_1hr_ **(E)** ^1%^PAox_3hr_ **(F)** ^1%^PAox_6hr_ *Denotes adduct peaks that do not correspond to actual oxidized antibody peaks.

**FIGURE 3:**
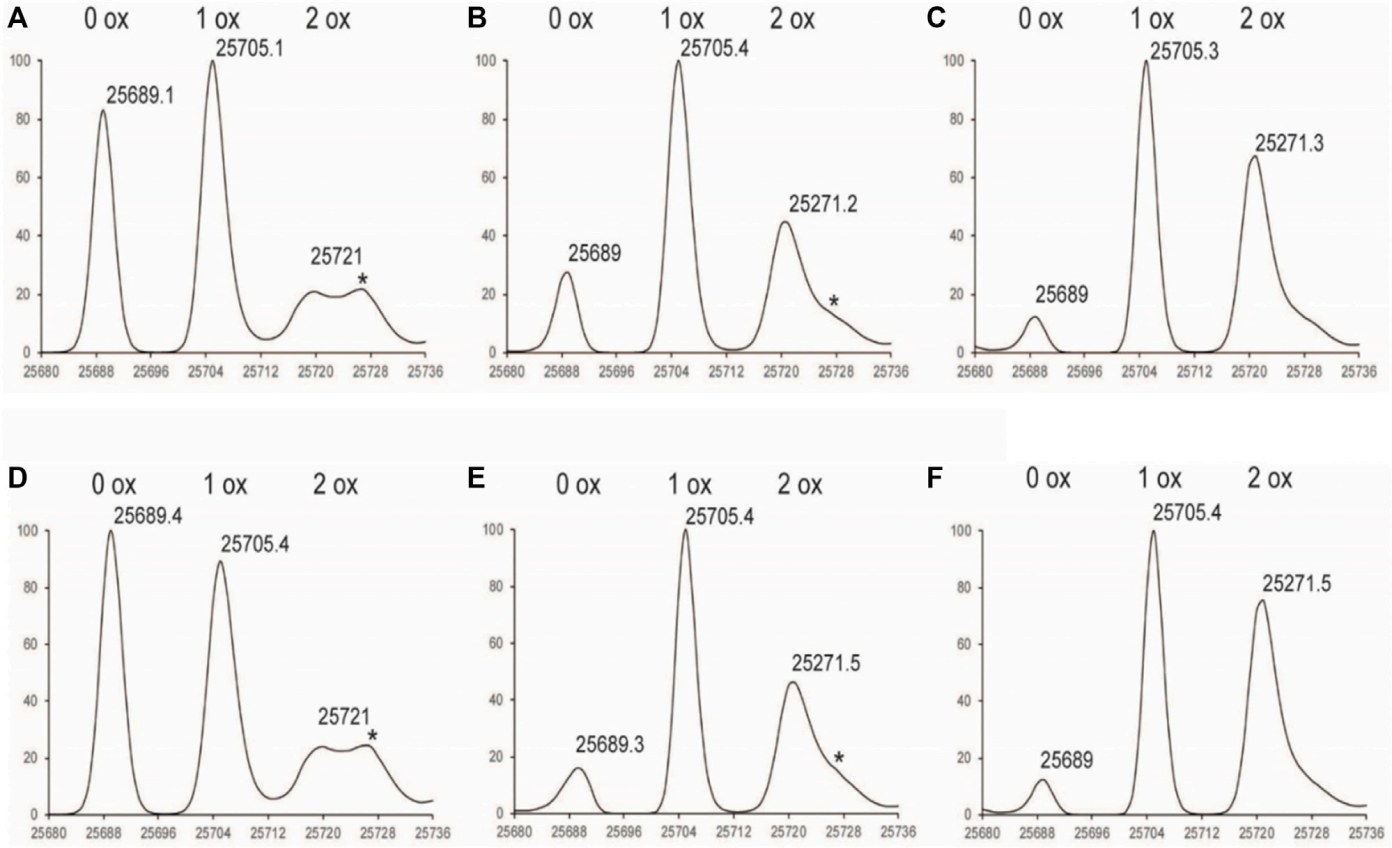
Deconvoluted LC-MS Spectrum of Fd subunit **(A)** ^1%^Nox_1hr_ **(B)** ^1%^Nox_3hr_ **(C)** ^1%^Nox_6hr_ **(D)** ^1%^PAox_1hr_ **(E)** ^1%^PAox_3hr_ **(F)** ^1%^PAox_6hr_ *Denotes adduct peaks that do not correspond to actual oxidized antibody peaks.

Exposure to H_2_O_2_ resulted in up to six methionine oxidation events observed on the NISTmAb: three events in the Fc region, one in the LC, and two in the Fd subunit (Figures 1–3). Each glycoform of the Fc was observed to oxidize at the same rate, therefore the mass range was zoomed in to focus on the G0F glycoform for easier visualization in Figure 1. As the Fc methionine residues oxidize over time in the Nox samples, four distinct species can be detected corresponding to 0, 1, 2, and 3 oxidized methionine residues with each oxidation event adding +16 da (Figures 1A–C). Longer time courses resulted in a larger relative abundance of scFc containing more oxidation events. The oxidation profile of the scFc of PAox samples showed substantially less oxidation than Nox at all time points (Figures 1D–F).

The oxidation profiles of the LC and Fd subunits of Nox and PAox samples, however, were quite similar and showed nearly the same oxidation levels at each timepoint (Figure 2, Figures 3A–C vs. Figures 3D–F). The LC subunit shows two distinct species corresponding to 0 and 1 oxidized methionine while the Fd subunit shows three distinct species corresponding to 0, 1, and 2 oxidized methionine residues (Figure 2, Figures 3A–C). The IdeS subunit analysis of these samples indicate that methionine oxidation kinetics of the Fc region of NISTmAb due to H_2_O_2_ exposure can be significantly slowed when protein A is added to the solution. Presumably the protein A remains bound to NISTmAb Fc to a sufficient degree, blocking putative oxidation sites, while the oxidation rates in the Fab region remain relatively unchanged.

While our in solution oxidation results were promising, the solution phase protection method had a few limitations leading to a final product that was not ideal for further downstream studies:1) protein A was still in solution and while bound to NISTmAb cannot easily be removed 2) protein A is a multidomain protein that has five different domains that all bind IgG Fc with varying degrees (Ljungberg et al., 1993) and 3) some protein A domains have also been shown to bind to the Fab region. To address these issues, another strategy was developed that takes advantage of a commercially available resin, MabSelect SuRe, which contains an engineered protein A covalently conjugated to agarose matrix. The MabSelect SuRe resin contains a tetramer of a Z domain, an alkali tolerant mutant of the B domain of protein A, which has multiple properties that address previously mentioned drawbacks of the free in solution oxidation method: 1) the protein A is attached covalently to the resin and has low ligand leaching (GE Healthcare, 2005) 2) the use of only the Z domain has less affinity and selectivity variation vs. the multi-domain protein A and 3) the Z domain has been shown to have little to no Fab binding (Jansson et al., 1998). On column oxidation was therefore pursued for the generation of samples with unique oxidation profiles when compared to traditional solution phase oxidation without protein A.

### Generation of Selectively Oxidized NISTmAb Samples and Comparison to Standard Forced Oxidized Time Course Samples Using LC-MS/MS Analysis

After the in solution proof of principle studies and the rationale for a more optimized approach, a larger scale study was performed to generate both standard forced oxidized samples along with a set of uniquely oxidized samples generated by oxidation on a protein A column for comparison purposes. Traditional accelerated forced oxidized samples (no protein A) with a wide range of total oxidation were generated using conditions known to produce materials with low levels of oxidation all the way to near complete oxidation of all susceptible methionine residues. A lower H_2_O_2_% was used, 0.3%, so that functionally relevant materials with very low levels of oxidation could be generated. Samples were stressed for a range of time points from 30 min to 32 h at 25°C focusing on early time points in the linear oxidation range and named according to the H_2_O_2_% and amount of time oxidized (^0.3%^Nox_0.5hr_, ^0.3%^Nox_1hr_, etc). A NISTmAb buffer exchange reference (NISTmAb Bex) was made without the addition of H_2_O_2_ but following a similar buffer exchange workup and NISTmAb control (NISTmAb Ctrl) was made without the addition of H_2_O_2_ but a 32 h incubation at 25°C to control for any changes that might occur over time. The exact conditions are detailed in the materials and methods. Another set of selectively oxidized samples was also generated by performing the forced oxidation while NISTmAb was bound to a protein A column. Specifically, three samples were generated using the same 0.3% H_2_O_2_ to allow for a direct comparison to time course stressed samples: 2, 4, and 6 h and named accordingly ^0.3%^CPA_2hr_,^0.3%^CPA_4hr_, ^0.3%^CPA_6hr_. Two other selectively oxidized samples were generated to evaluate on/off equilibrium effects: one with gentle stress over a long time period (0.0375% H_2_O_2_ for 16 h), ^0.0375%^CPA_16hr_ and another with heavy stress for a short time period (3% H_2_O_2_) for 1 h, ^3%^CPA_1hr_. The exact conditions of all on column oxidized samples are detailed in the materials and methods.

**FIGURE 4:**
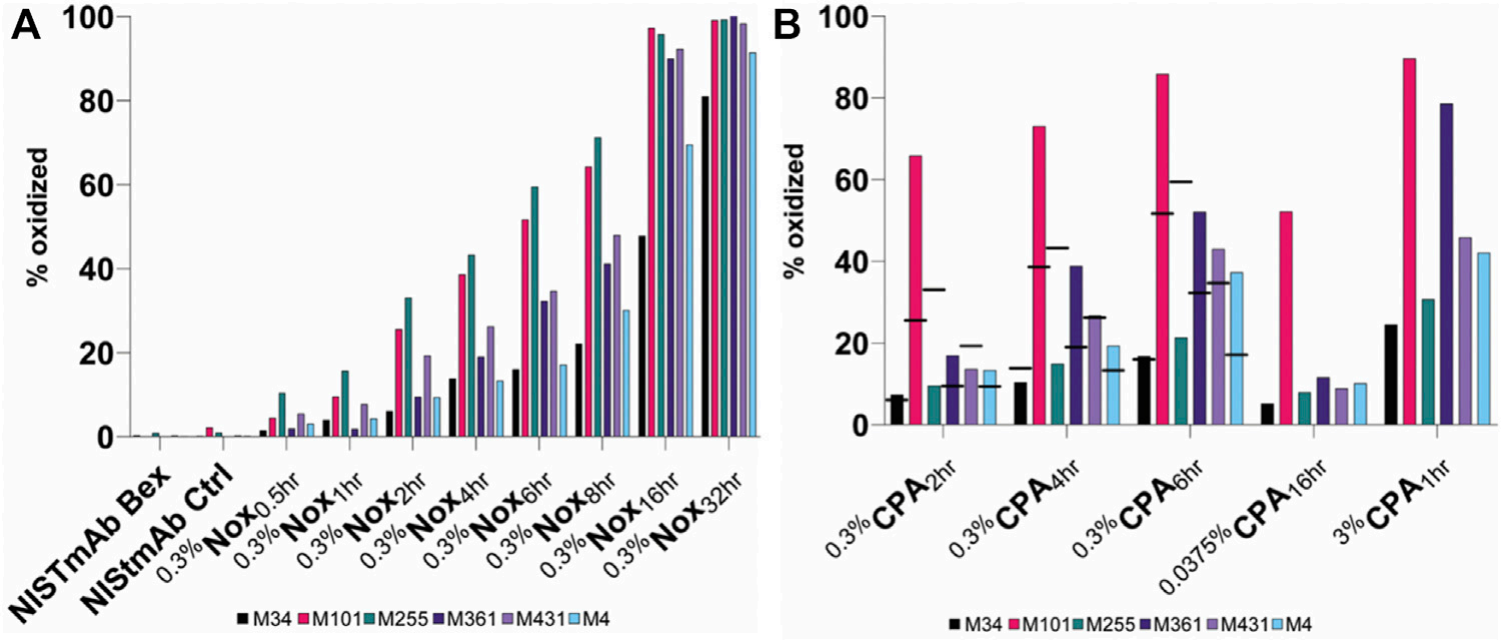
Relative abudance of oxidized methionine residues in NISTmAb samples **(A)** NISTmAb Bex and Ctrl with Nox Samples **(B)** CPA samples. Black dashes (−) are used in the 0.3% CPA samples to denote the relative abundance of each methionine residue from equaivlanet Nox sample for comparison purposes.

To demonstrate site specific changes in the oxidation profile of on column stressed material, tryptic peptide mapping combined with liquid chromatography mass spectrometry (LC-MS) was performed. Following peptide identification by tandem mass spectrometry, the extent of quantifiable methionine oxidation was determined by quantitative evaluation of the modified tryptic peptides relative to their respective unmodified parent peptides as described in methods section. All peptides containing a given Met residue were collated to provide the residue-specific quantification results. Among the eight methionine residues, only six changed significantly under our ^0.3%^Nox accelerated stress conditions: HC M34, M101, M255, M361, M431 and LC M4 as summarized in Figure 4A. The levels of other typical modifications (deamidation, isomerization, glycation) were monitored and found that no other significant modifications occurred in any of the ^0.3%^Nox samples. The ^0.3%^Nox samples showed an increase in oxidation over time (Figure 4A). Typically, methionine residues in the Fc are known to be the most susceptible to oxidation while methionine residues in the variable regions of the Fab vary in their susceptibility to oxidize depending on a variety of factors including solvent-accessibility of the methionine residue, expression host, and process conditions (Yang et al., 2017). Specifically, the oxidation rates of susceptible methionine residues in NISTmAb ordered as the following: M255 > M101 > M431 > M361 > LC M4>M34. The oxidation profile of ^0.3%^Nox samples can be generalized with three tiers of oxidation with the first and most susceptible being M255 and M101, followed by M431 and M361 s, and third and least susceptible being M34 and LC M4.

M255 was oxidized at much lower rate in the ^0.3%^CPA samples when identical timepoints were considered (indicated by black dashes in Figure 4B), implying that when NISTmAb is bound to protein A, M255 is protected from oxidation. In addition to the dramatic protection observed for M255 oxidation, a slight decrease (and hence minor protection) in oxidation at M431 was also shown in the ^0.3%^CPA_2hr_ sample but not seen in the 4 and 6 h samples. While a direct comparison in the ^0.3%^CPA_4hr_ and ^0.3%^CPA_6hr_ samples did not show lower oxidation at M431 it was clear that oxidation was lower at M431 than M361 in CPA samples but in Nox samples the opposite was true so oxidation in the presence of protein A did seem to have a slight protective effect on M431 as well. This phenomenon is in agreement with the known relative solvent exposure of protein A-bound IgG1; protein A is known to be in close contact with M255 while M431 is also nearby (Deis et al., 2015). Interestingly, a higher rate of oxidation at M101 (and to a lesser degree M361 and LC M4) was observed when compared to other residues. This could indicate a conformational change of the Fab when IgG is bound to protein A that makes M101 more accessible and/or an orientational effect while ligand-bound. Specifically, the oxidation rates of susceptible methionine residues in CPA samples ordered as the following: M101 > M361 > M431 > LC M4>M255 > M34. The oxidation profile of CPA samples can be generalized with three tiers of oxidation with the first and most susceptible being M101, followed by M361, M431, and LC M4 second, and third and least susceptible being M34 and M255.

A similar phenomenon was seen in the ^0.0375%^CPA_16hr_ and ^3%^CPA_1hr_ samples with M255 showing a much slower oxidation rate relative to the other methionine residues when compared to standard forced oxidized ^0.3%^Nox samples; M255 displayed the fastest oxidation rate in Nox samples but in CPA samples it was much slower, second to last. ^0.0375%^CPA_16hr_ was shown to be a very interesting material with a high level of M101 oxidation (52%) while having only small amounts (<15%) of oxidation at all other sites. This material could be of interest to elucidate specific effects M101 oxidation might have on NISTmAb while minimizing the influence of other oxidation effects. ^3%^CPA_1hr_ was shown to be the most oxidized CPA sample, and the strategy of heavy stress for a short period did not seem to provide the same level of protection at M255 when compared to the milder conditions. Taken together, these measurements indeed confirm the ability of a ligand to mask a specific epitope from oxidation and the ability to generate uniquely oxidized challenge materials.

### Surface Plasmon Resonance: Assessment of NISTmAb Using Domain Specific Molecular Probes

**FIGURE 5:**
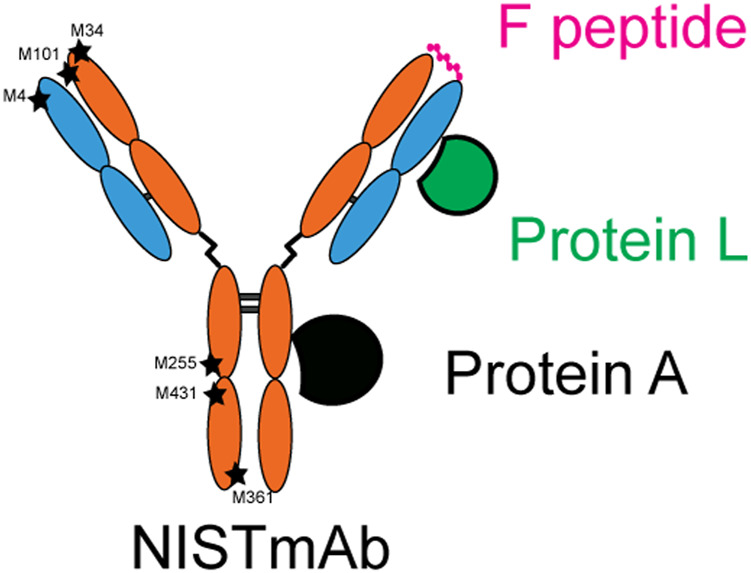
Diagram of molecular probes used in SPR assay and the relative location they bind.

Previous studies have shown that oxidation in both the Fab and Fc regions can affect binding to both antigen and Fc receptor targets. In order to characterize both domains and therefore potentially unique bioactivity of the novel challenge materials created herein, a surface plasmon resonance (SPR) assay was designed that could assess binding at distinct and separate locations using a set of NISTmAb binding proteins as molecular probes. Specifically, ligands were selected that are known to bind in both the Fab and Fc regions so functionality of both domains could be assessed. Two common bacterial proteins were selected that are known to have a high affinity for NISTmAb and have known binding sites: protein A which binds to the Fc and protein L which binds to the variable region of the LC Fab without interfering with antigen binding site. A peptidic epitope, F peptide, known to bind with high affinity to the NISTmAb CDR of the Fab was also included. A schematic diagram showing the three molecular probes and the relative location they bind to NISTmAb is shown in Figure 5. A reversible biotin capture system was used for each of the ligands that has been shown to be an efficient SPR assay platform that allows for a generic regeneration condition and repeatable capture (Karlsson et al., 2018). The assay depends on a special sensor chip CAP which has a pre-immobilized oligonucleotide on the surface. Sequential steps of the assay include: 1) A biotin capture reagent (streptavidin modified with the complementary oligonucleotide) is hybridized to the CAP chip, 2) The biotinylated ligand (protein A, protein L, or F peptide) is captured by streptavidin, 3) the adsorption/desorption of the analyte is observed, and 4) finally the sensor chip surface is completely regenerated back to the bare oligonucleotide (Supplementary Figure S2).

A unique target of the current assay was to demonstrate that reliable and quantitative differentiation of the binding could be achieved with a single replicate of material and sole observation of the maximum response level as opposed to detailed concentration-dependent equilibrium and/or kinetic fitting models. Repeatability of this platform (additional details in materials and methods) was demonstrated using 20 consecutive cycles of ligand capture and NISTmAb Bex as the analyte. Response levels for each of the biotinylated ligands were repeatable with coefficient of variations (CV) of less than 3.4% (Supplementary Table S2). Very repeatable ligand capture levels are vital for assay performance and were achieved by the addition of 0.1% bovine serum albumin to both protein A and protein L solutions but not needed in the F peptide solution. The binding response for each NISTmAb Bex replicate was also confirmed to be repeatable with CVs of less than 3.3% (Supplementary Table S3). These results indeed confirmed that the assay was repeatable and that this assay could provide reliable data on our oxidized sample set. The final experimental design for future use therefore consisted of 20 cycles with 3 startup cycles to prep the surface, 3 NISTmAb Bex samples run at the beginning, middle, and end, and 14 samples run once in a randomized order. Samples were all diluted to 200 nmol/L concentration and run over once per experiment. Each experiment was repeated 3 times providing 3 measurements for each oxidized sample and nine measurements of NISTmAb Bex. After analysis of all samples, the repeatability and reproducibility of the assay was also confirmed by measuring the response level in response units (RU) for each of the biotinylated ligands after each cycle. All three ligands also showed intra assay CV less than 2% and inter assay CV of less than 5% (Supplementary Table S4–6). All oxidized samples were characterized by size exclusion chromatography (SEC) to ensure that samples did not have any substantial changes in high molecular weight (HMW) or low molecular weight (LMW) species that would have interfered with SPR and thermal unfolding studies. The HMW, monomer, and LMW species were determined for each sample and summarized in Supplementary Table S7. Overall, all the oxidized samples except ^0.0375%^CPAox_16hr_ showed only minor differences when compared to NISTmAb Bex and the small difference in that one sample was noted but was not considered an issue for further biophysical studies.

### Surface Plasmon Resonance: Effects of Methionine Oxidation on NISTmAb Binding to Domain Specific Molecular Probes

#### General Trends of Nox Samples

**FIGURE 6:**
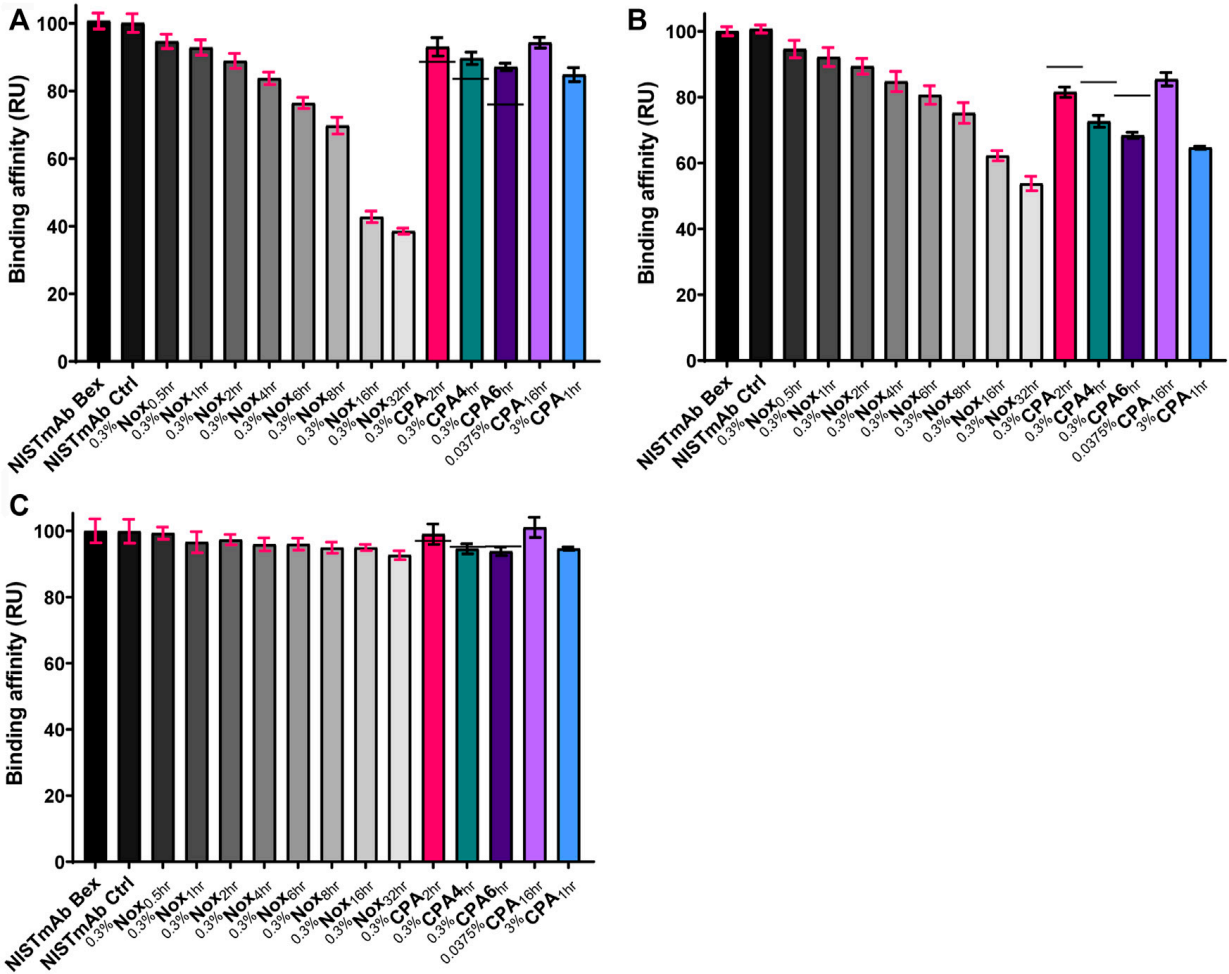
Relative binding affinity of NISTmAb samples: **(A)** Protein A binding affinity (RU) **(B)** F peptide binding affinity (RU) **(C)** Protein L binding affinity (RU). The standard deviation of each measurement is noted with error bars. Black dashes (−) are used in the 0.3% CPA samples to denote the relative binding affinity from equivlent timed Nox sample for comparison purposes.

The relative binding affinity of oxidized NISTmAb samples binding to protein A can be seen in Figure 6A and the representative sensorgrams in Supplementary Figures S3A,B. A significant decrease in relative binding affinity correlating to increasing oxidation levels can be quickly identified when looking at ^0.3%^Nox time course samples. The least oxidized sample, ^0.3%^Nox_0.5hr_, showed relatively small changes (94.7%) in binding to protein A relative to NISTmAb Bex, whereas the ^0.3%^Nox_32hr_ sample, which is the most oxidized sample with near complete oxidation of the six methionine residues shown to oxidize under our conditions, showed a drastic decrease in the relative binding affinity to protein A (38.5%). The F peptide binding results showed a similar trend of decreasing relative binding affinity with increasing global oxidation as shown in Figure 6B and the representative sensorgrams in Supplementary Figures S3C,D. The least oxidized sample, ^0.3%^Nox_0.5hr_, showed relatively small changes (94.6%) in binding to F peptide relative to NISTmAb Bex. On the other side of the time course scale, the ^0.3%^Nox_32hr_ sample, which is the most oxidized sample with near complete global oxidation, showed large changes to the relative binding to F peptide (53.7%). While the protein A and F peptide binding data showed major changes due to oxidation, the protein L binding data showed very little changes as oxidation increased (Figure 6C) (Supplementary Figures S3E, F). The least oxidized sample, ^0.3%^Nox_0.5hr_, showed no real changes (99.3%) in binding to protein L relative to NISTmAb Bex. Even the near completely oxidized ^0.3%^Nox_32hr_ sample showed only a small change in relative binding to protein L (92.6%).

#### General Trends of CPA Samples

The relative binding affinity of ^0.3%^CPA samples binding to protein A, F peptide, and protein L can also be seen in Figures 6A–C, respectively. The binding level of identical time points of ^0.3%^Nox samples is shown with a solid line in each figure. The relative binding affinity of ^0.3%^CPA samples to protein A was higher when compared to identical time points of ^0.3%^Nox samples, implying that protection from oxidation due to the protein A masking did indeed help maintain rebinding to a protein A ligand. This phenomenon, while interesting, was expected as M255 has been shown to be vital for high affinity protein A binding and was shown to be protected from oxidation in the 0.3 %CPA samples.

Protein A masking had little to no effect on protein L binding as indicated in Figure 6C. This may be the expected result considering protein A and protein L binding regions are spatially distributed as demonstrated in Figure 5, and no methionine residues of IgG are known to be involved in protein L binding, a supposition supported by our observation of little to no change in Nox sample binding to protein L. Protein A masking did, however, have an effect on F peptide binding (Figure 6B). The relative binding affinity of ^0.3%^CPA samples to F peptide was lower when compared to identical time points of ^0.3^^%^Nox samples, implying that the changes in oxidation rates due to the protein A masking did affect F peptide binding. Qualitative comparison of Figure 4B reveals that M101 had the largest increase in oxidation vs. the equivalent Nox time points. A correlation would make sense considering M101 is located in the CDR and therefore probable to have some effect on antigen binding. On the other hand, M361 and LC M4 are also consistently more oxidized in the 0.3 %CPA samples, making a conclusive correlation subjective at best without epitope mapping and of course neglecting the possibility of allosteric effects.

#### Site-Specific Correlation: % Met Oxidized vs. Relative Binding

**FIGURE 7:**
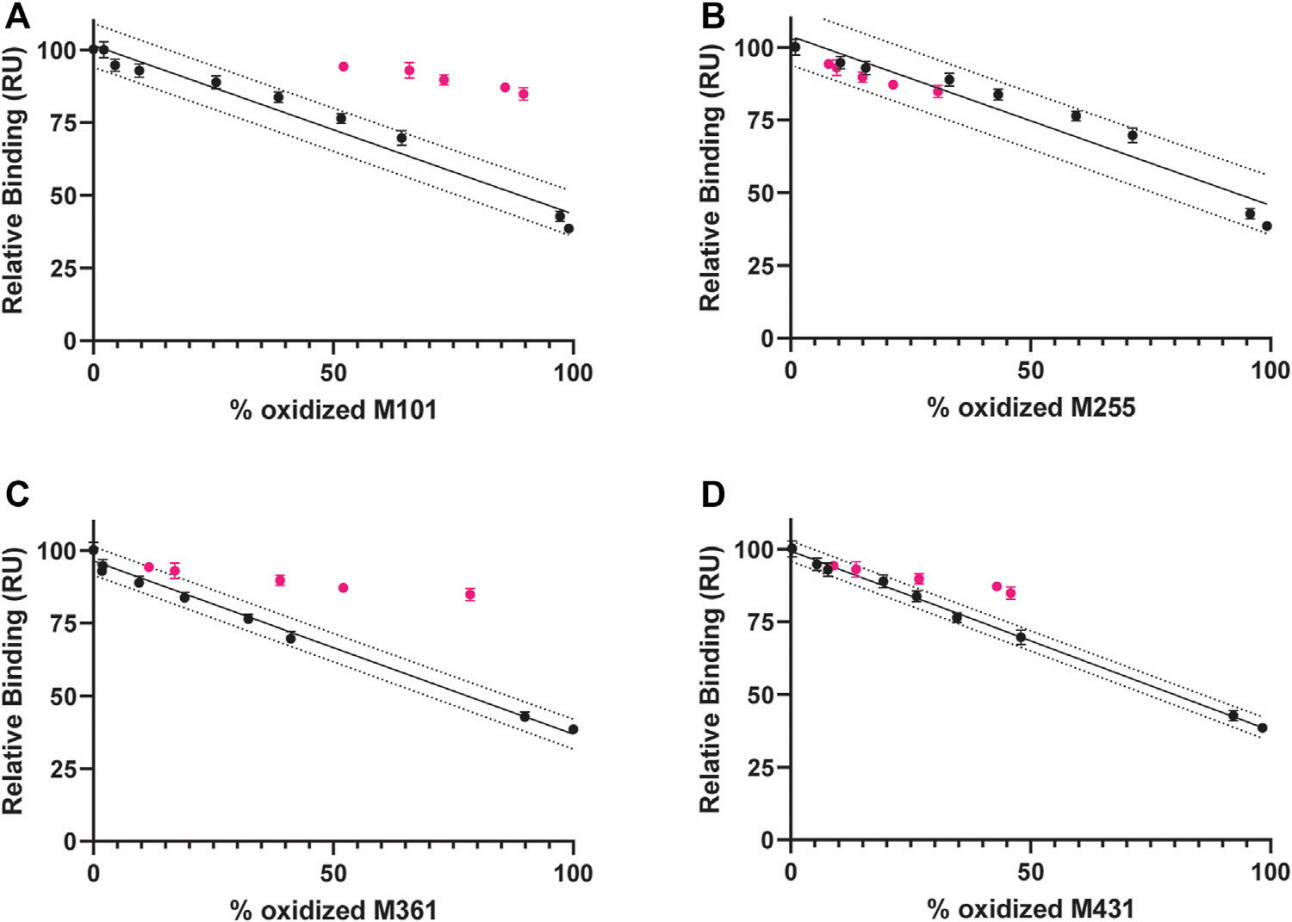
Correlation between protein A binding and Met ox (^0.3%^Nox samples in black and CPA samples in pink) and linear regression analysis of Nox samples (solid black line) with 90% prediction bands (dotted black line) **(A)** M101 **(B)** M255 **(C)** M361 **(D)** M431.

Peptide mapping analysis was used to evaluate oxidation at specific methionine residues and combined with relative binding affinity by SPR to further explore site-specific structure/function correlations. Figure 7 A through D shows a consistent trend for Met residues in the ^0.3%^Nox samples (in black), an increase in oxidation at each site appears to correlate with a decrease in protein A relative binding. Based on these samples alone, however, no confident site-specific correlations can be made because oxidation at all methionine residues is increasing and roughly correlating with a decrease in protein A binding. Inclusion of the CPA samples (in pink), however, allow for a more selective and confident site-specific relationship because oxidation rates at some residues remain unaltered, while others change as a result of ligand protection. CPA samples for M101 and M361, for example, show a higher relative binding at the same % oxidation (Figures 7A,C) and these data stay widely from the ^0.3^^%^Nox regression line and fall completely out of 90% prediction bands. These data indicate oxidation at these sites is not the dominant driver of protein A binding reduction. However, CPA samples for M255 and M431 trend more closely to the ^0.3^^%^Nox regression line (Figures 7B,D), indicating decreased protein A binding is more closely associated with oxidation at these sites. Previous reports have indeed demonstrated that oxidation in the Fc region disrupts protein A binding as it binds to a region where M255 and M431 are located and where the C_H_2 and C_H_3 domains interact (Deis et al., 2015; Gallagher et al., 2018). In the above example, it is our position that if a specific residue is a causal factor in reducing binding, then alteration of the kinetics of that oxidation (e.g., slowed *via* ligand-bound protection) will not alter the trend of relative binding vs. site-specific %Met oxidation. Specifically, CPA data points will deviate farther from the Nox regression model when residues are not involved in binding while CPA data points will trend closer to the Nox regression model when they are involved in binding. To quantitate this distance, the standard error of regression (Sy.x) of CPA data points was calculated from the Nox regression model. The Sy.x values are shown in Supplementary Table S8 and show significantly larger values for M101 and M361, more intermediate value for M431, and lowest for M255. In summary, oxidation at M255 correlates strongly to decreased protein A binding while oxidation at M431 might have a minor secondary effect and oxidation at M101 and M361 seem to play no direct role in decreased protein A binding.

A similar trend can be seen in Supplementary Figures S4 A–D for Met residues in the ^0.3%^Nox samples, an increase in oxidation at each site appears to correlate with a decrease in F peptide relative binding. More selective and confident correlations are achieved when the CPA samples are added to these plots. CPA samples for M34 and M255 show a lower relative binding to F peptide at the same % oxidation and these data stay widely from the ^0.3^^%^Nox regression line and fall completely out of 90% prediction bands (Supplementary Figures. S4 C,D). CPA samples for LC M4 and M101, on the other hand, trend more closely to the Nox regression line (Supplementary Figures S4 A,B). Again the Sy.x was calculated and values are shown in Supplementary Table S9. The values are significantly higher for M34 and M255, more intermediate for LC M4, and lowest for M101. In summary, oxidation at M101 correlates strongly to decreased F peptide binding while oxidation at LC M4 might have a minor secondary effect and oxidation at M34 and M255 seem to play no direct role in decreased F peptide binding. Previous reports have shown that the oxidation of a residue in the CDR of an antibody can disrupt antigen binding (Haberger et al., 2014), and the current F peptide binding data indicates that methionine oxidation can indeed cause a significant decrease in the ability of NISTmAb to bind to a peptide antigen.

### Surface Plasmon Resonance: Sensitivity to Detect Changes in Oxidation

To determine what samples had statistical differences in the binding affinity when compared to the unstressed NISTmAb Bex sample, an unpaired *t*-test was used to calculate the differences between the ^0.3%^Nox samples and the NISTmAb Bex sample (Supplementary Table S10). First, any differences due to storage at room temperature for an extended period of time were ruled out by demonstrating that the binding of NISTmAb Bex and NISTmAb Ctrl were not statistically different. Looking next at the least oxidized sample, ^0.3%^Nox_0.5hr_, it did indeed show significant differences in binding to protein A when compared to NISTmAb Bex with a corresponding *p* value of 0.0028. All other samples were also statistically different than NISTmAb Bex with *p*-values of less than 0.0001. An unpaired *t*-test was again used to determine the statistical difference in relative binding affinity to F peptide. The binding of NISTmAb Bex and NISTmAb Ctrl were not statistically different when binding to F peptide which confirms that no modifications due to storage at 25°C for 32 h led to any detectable changes. Again the least oxidized Nox sample, ^0.3%^Nox_0.5hr_, could be differentiated from NISTmAb Bex with F peptide binding as the relative binding affinity was statistically different with a *p* value of 0.0008. All other samples were also statistically different than NISTmAb Bex with *p*-values of less than 0.0001. A summary of *p* values for all samples can be found in Supplementary Table S8. Taken together both protein A and F peptide binding are good indicator of oxidation and even small amounts of oxidation could be detected using this approach.

The sensitivity of detection of specific oxidized methionine residues in NISTmAb could be estimated by using the experimental data from our least oxidized sample, ^0.3%^Nox_0.5hr_. protein A binding was indicative of methionine oxidation at M255 and the sensitivity of detection was estimated to be 10% of M255 oxidation from experimental data of sample ^0.3%^Nox_0.5hr_. F peptide binding was indicative of methionine oxidation at M101 and the sensitivity of detection was estimated to be 5% of M101 oxidation from experimental data of sample ^0.3^^%^Nox_0.5hr_.

Overall the trends for the traditional Nox samples were as expected—higher global oxidation, indicated increase in oxidation at all susceptible Met residues (Figure 4A), resulted in decreased binding for oxidation-affected interactions. In general, oxidation affects NISTmAb binding to protein A > F peptide and only the smallest amount for protein L. The general trend also held for the CPA samples, oxidized samples demonstrated lower binding affinity compared to NISTmAb Bex. Both protein A and F peptide make excellent molecular probes to distinguish methionine oxidation in NISTmAb as they can detect only minor changes in oxidation but protein L was unable to detect these minor changes. This SPR assay has been demonstrated to be a rapid method to detect oxidation at both the Fc and Fab regions in the same analytical run.

### Assessment of NISTmAb Stability With Thermal Unfolding Assay

In the discussion above we utilized the uniquely oxidized CPA samples to demonstrate that localized changes in oxidation would inevitably affect ligand binding in that region. The SPR assay developed was therefore inferred to be sensitive to oxidation of specific Met residues. A logical question to then ask is whether other biophysical assays indicative of stability may also be localized using similar techniques. Qualitative thermal melting studies with intrinsic tryptophan fluorescence were therefore performed to identify correlations between methionine oxidation and domain stability. Oxidation of IgG is known to destabilize the Fc domain of IgG resulting in changes in the melting temperature (Chumsae et al., 2007; Gao et al., 2015). Previous differential scanning calorimetry (DSC) measurements of NISTmAb have determined that there are three distinct melting temperatures, 69.2°C, 83.1°C, and 93.4°C, corresponding to C_H_2, C_H_3, and Fab domains (Gokarn et al., 2015). To examine the thermal stability of stressed samples in the current study, a thermal unfolding assay was performed that measures the intrinsic fluorescence from aromatic amino acid residues. Changes in the fluorescence ratio 350 nm/330 nm were measured with increasing temperature, and the midpoint unfolding inflection temperatures (Ti) were calculated along with the initial 350 nm/330 nm ratio.

**FIGURE 8:**
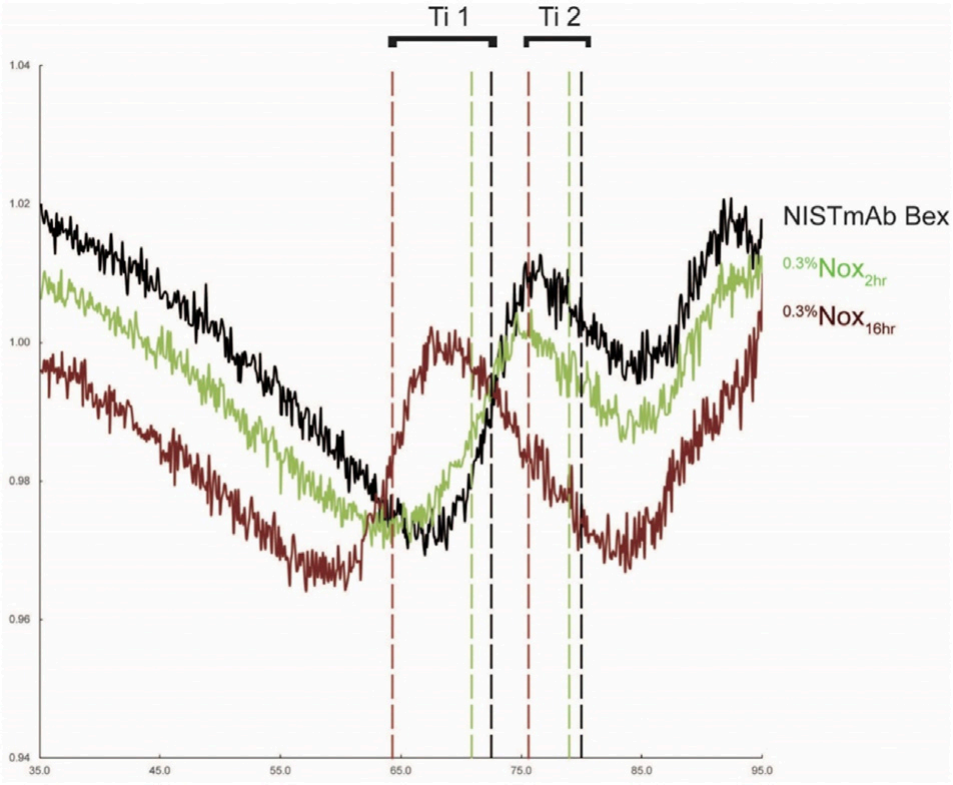
Unfolding profiles of NISTmAb Bex, a lightly oxidized sample (^0.3%^Nox_2hr_), and a heavily oxdized sample (^0.3%^Nox_16hr_) with calculated inflection temperatures for the C_H_2 (Ti_1_) and C_H_3 (Ti_2_) domains (.

**FIGURE 9:**
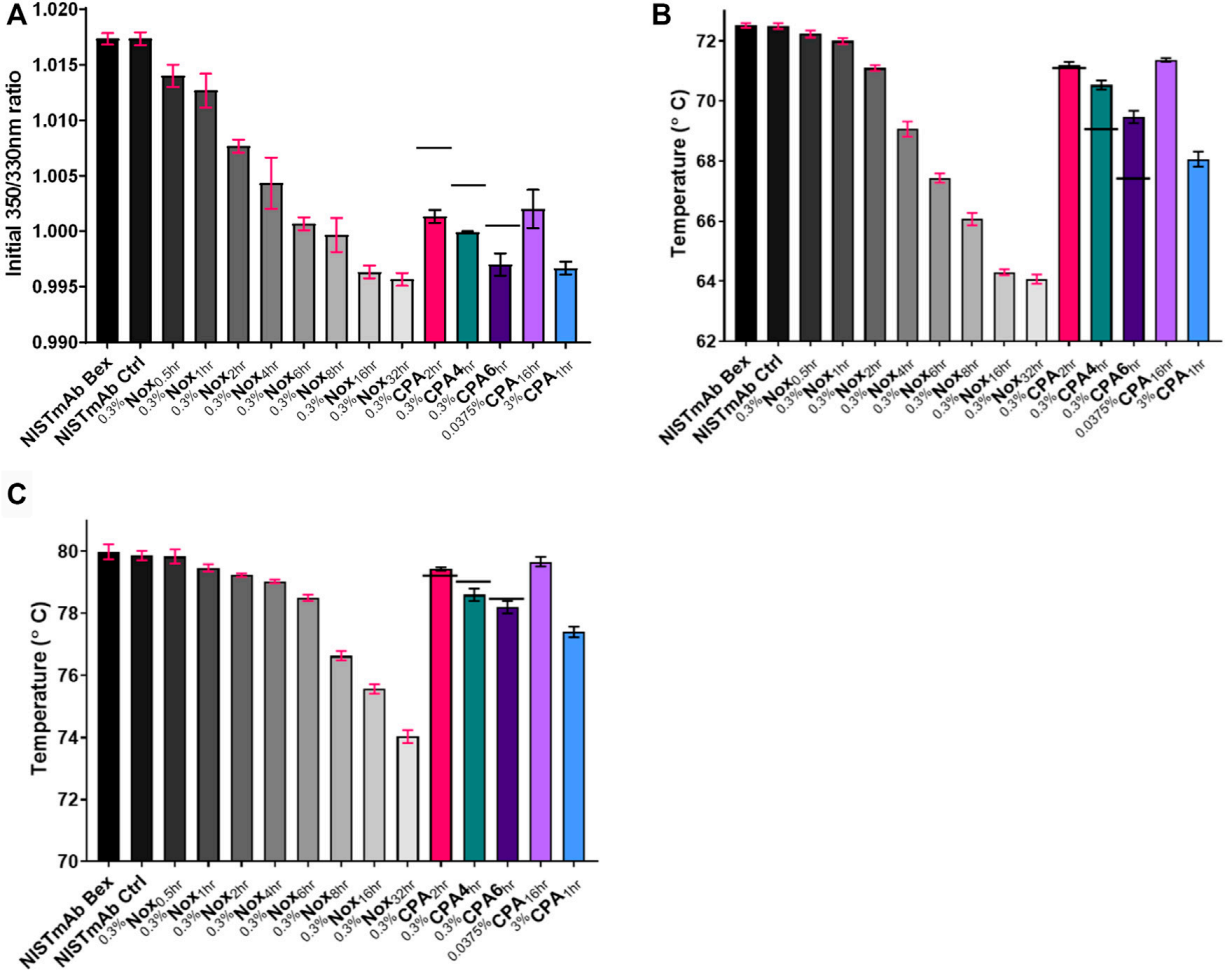
Thermal unfolding analysis using Tycho NT 6.0 **(A)** Initial 350 nm/330 nm ratio **(B)** Inflection Temperature 1 **(C)** Inflection Temperature 2. The standard deviation of each measurement is noted with error bars. Black dashes (−) are used in the 0.3% CPA samples to denote the relative binding affinity from equivlent timed Nox sample for comparison purposes.

Representative unfolding profiles of NISTmAb Bex, a lightly oxidized sample (^0.3%^Nox_2hr_), and a heavily oxidized sample (^0.3%^Nox16_hr_) are shown in Figure 8. These unfolding profiles showed two clear changes that relate to the level of oxidation. First, the initial ratio of detected fluorescence signal decreases while oxidation increases indicated by the downward shift of the profile at 35°C (the beginning of the assay measurement). This decrease signifies a change in the amount of solvent exposed tryptophan or tyrosine residues. Second, the thermal stability of oxidized samples decreased as methionine oxidation increased indicated by the leftward shift (decreasing value) in both Ti_1_ and Ti_2_. Three distinct Ti’s could be determined for NISTmAb Bex: 72.5°C (Ti_1_), 80.0°C (Ti_2_), and 89.1°C (Ti_3_). The software was unable to determine Ti_3_ for all samples, however manual inspection of the first derivative of the raw data shows Ti_3_ was similar for all oxidized samples. The complete results of the unfolding measurements including the initial ratio and unfolding temperatures for all samples can be found in Figure 9.

#### General Trends of Nox Samples

When looking over the thermal unfolding data it is evident that as oxidation increases the initial ratio and both unfolding temperatures decrease significantly. A small decrease in the initial ratio can be seen in Figure 9A correlating to increasing oxidation levels when looking at ^0.3%^Nox time course samples. The decrease of the initial ratio is small in value, 0.003, for the least oxidized sample, ^0.3%^Nox_0.5hr_, and 0.021 for the most oxidized sample. While these values are indeed small, the measurement of the initial ratio was shown to be extremely precise with the average coefficient of variation of 0.09% indicating that even small differences could be reliably measured.

A large decrease in the Ti_1_ can be seen in Figure 9B correlating to increasing oxidation levels when looking at ^0.3%^Nox time course samples. The least oxidized sample, ^0.3%^Nox_0.5hr_, showed a small decrease in Ti_1_ of 0.3°C relative to NISTmAb Bex. Whereas, the ^0.3%^Nox_32hr_ sample, the most oxidized sample, showed a drastic decrease in Ti_1_ of 8.45°C. Similarly, a large decrease in the measured Ti_2_ can be seen in Figure 9C correlating to increasing oxidation levels when looking at ^0.3%^Nox time course samples. The least oxidized sample, ^0.3%^Nox_0.5hr_, showed a small decrease in Ti_2_ of 0.15°C relative to NISTmAb Bex. Whereas, the ^0.3^^%^Nox_32hr_ sample, the most oxidized sample, showed a substantial decrease in Ti_1_ of 5.95°C.

#### General Trends of CPA Samples

The initial ratio and thermal unfolding temperatures of ^0.3^^%^CPA samples can also be seen in Figures 9A–C, respectively. The measured initial ratio/unfolding temperature of identical time points of ^0.3%^Nox samples is shown with a solid line in each figure for comparison purposes. The initial ratio of ^0.3%^CPA samples was much lower when compared to identical time points of ^0.3%^Nox samples, implying that changes in the methionine oxidation pattern due to the protein A masking did decrease the initial ratio. This significant decrease in initial ratio was seen in heavily oxidized Nox samples suggesting that oxidation of M101 is likely to be the cause of the change as it is heavily oxidized in all CPA samples. This phenomenon is confirmed when comparing the other CPA samples especially ^0.0375%^CPA_16hr_ which has very low levels of oxidation at all sites except M101. ^0.0375%^CPA_16hr_ has a similar amount of oxidation at M101 as ^0.3%^Nox_6hr_ and these samples indeed have similar initial ratio values, 1.002 and 1.001 respectively. Taken together oxidation at M101 seems to have a strong effect on the initial ratio and oxidation at this site seems to induce a structural change that exposes a tyrosine or tryptophan residue likely in the Fab region.

Protein A masking also had an effect on the measured Ti_1_ (Figure 9B). The Ti_1_ of ^0.3%^CPA samples was much higher at time points 4 h and 6 h but essentially the same at 2 h when compared to identical time points of ^0.3%^Nox samples. This implies that changes in the oxidation rates due to protein A masking did increase the stability of the C_H_2 domain by blocking oxidation. Presumably, this effect is mostly due to the much lower amount of oxidation at M255 in CPA as it is the most protected residue and also is located in the C_H_2 domain. While the initial ratio and Ti1 values were significantly affected by protein A masking oxidation, the value of Ti_2_ of ^0.3%^CPA samples was slightly higher at 2 h and slightly lower at 4 and 6 h compared to ^0.3%^Nox samples (Figure 9C). The changes in oxidation rates due to the protein A masking had a smaller effect on the stability of the C_H_3 domain of NISTmAb. This effect is most likely due to the higher amounts of oxidation at M361 and M431 in the CPA samples both of which are located in the C_H_3 domain of NISTmAb.

#### Site-Specific Correlation: % Met Oxidized vs. Initial Ratio/Unfolding Temperatures

Peptide mapping analysis was used to evaluate oxidation at specific methionine residues and combined with thermal unfolding analysis to further explore site-specific structure/function correlations. Plots of the initial ratio of the ^0.3%^Nox samples against the % oxidized of methionine at all sites were generated and nonlinear regression analysis was used to model Nox data sets (Supplementary Figures S5A–F). A consistent trend can be seen for all Met residues in the Nox data, an increase in oxidation at each site correlates with a decrease in the value of the initial ratio. More selective and confident correlations are achieved when the CPA samples are added to these plots. CPA samples for M34, M255, and M431 stay widely from the ^0.3%^Nox regression model and fall completely out of 90% prediction bands (Supplementary Figures S5B, D, F). On the other hand, CPA samples for LC M4, M101, and M361 trend more closely to the Nox regression model (Supplementary Figures S5A,C,E). The Sy.x of CPA data was calculated and the values are shown in Supplementary Table S11. The Sy.x was significantly higher for M34, M255, and M431, more intermediate for LC M4 and M361, and lowest for M101. In summary, oxidation at M101 correlates strongly with the decrease in initial ratio, while oxidation at LC M4 and M361 could play a more secondary role. Methionine oxidation at these sites seem to induce structural change that causes a tyrosine or tryptophan residue to be less exposed, resulting in a shift of the fluorescence toward lower wavelengths.

Strong correlating trends can be seen when Ti_1_ is plotted vs. % of methionine oxidation in the Nox samples (Supplementary Figures S6A–F). Again, CPA samples were added to these plots to get a more precise idea of which oxidized methionine residues play a stronger role in the decrease of thermal stability of NISTmAb. CPA samples for LC M4, M101, and M361 stay widely from the ^0.3%^Nox regression model and fall completely out of 90% prediction bands (Supplementary Figures S6A,C,E). On the other hand, CPA samples for M34, M255, and M431 trend more closely to the Nox regression model (Supplementary Figures S6B,D,F). The Sy.x of the CPA data was calculated and the values are shown in Supplementary Table S12. The Sy.x was significantly higher for LC M4, M101, and M361, more intermediate for M34 and M431, and lowest for M255. These data indicate that oxidation at M255 correlates strongly with the decrease in thermal stability of the CH2 domain, while oxidation at M34 and M431 could play more secondary roles.

Similar strong correlations are observed after plotting Ti_2_ vs. % of methionine oxidation in the Nox samples (Supplementary Figures S7A–F). CPA samples for M101 stay widely from the Nox regression model whereas all others trend closely to their respective model. The Sy.x of the CPA data was calculated and the values are shown in Supplementary Table S13. The Sy.x was higher for M101 while all other methionine residues show relatively low values. These data indicate that global methionine oxidation at multiple residues contribute to the decrease in thermal stability of the CH3 domain.

## Perspectives

In order to evaluate methods that are capable of attribute specific monitoring, well-defined materials with site specific attribute modifications are needed. We explored a strategy that could generate selectively oxidized antibody materials with specific attribute changes that differ from global chemical oxidation. This ligand masking method takes advantage of the ability of a specific binding partner to bind and mask its binding region on an antibody. Once bound, an accelerated stress procedure was performed to generate a product with a unique methionine oxidation pattern. This approach generated materials that showed protection from oxidative stress at a specific methionine residue, M255, while other methionine residues were exposed to varying levels of oxidation. While this method still cannot provide perfect control over oxidation, unique materials with specific attribute modifications were generated that could not be made using a traditional approach. Other strategies can achieve similar unique materials; however, this method has some distinct advantages that make it an interesting approach. First, no genetic manipulations are needed that can mimic or block oxidation which can be arduous and require the expression and purification of new mAbs. Also these oxidation mimicking mutants (e.g., glutamine to mimic oxidized methionine) do share similarities in chemical structure and hydrophobicity. They are indeed different in structure, and the exact contribution still must be demonstrated. Another typical method relies on tedious chromatographic separations in combination with fractionation that can be difficult on a larger scale. In some cases oxidation variants can be almost impossible to separate without a very specialized affinity column.

Assigning criticality to a specific amino acid residue can be a challenging task due to the global nature of typical stress studies. While certain residues are typically modified at higher rates than others, multiple residues are most often modified, making correlations between a site-specific modification and biological activity difficult. Often multiple residues in both the Fab and Fc regions are susceptible to modifications, but the effect of each are difficult to differentiate. The ligand masking approach allowed generation of materials with unique modification patterns. The effect of this oxidation was explored by measuring changes in relative binding affinity and the thermal stability of these modified materials when compared to NISTmAb Bex. With the help of these uniquely stressed materials, specific attribute changes could be correlated to specific changes in these analytical assays; a pre-requisite to assigning attribute criticality with heightened specificity.

In this study, we explored the effect methionine oxidation has on the ability of NISTmAb to bind to three IgG binding proteins including protein A, an antigen mimic F peptide, and protein L. A SPR assay was developed that demonstrated the ability of these proteins to serve as molecular probes to rapidly reveal stress induced oxidation in NISTmAb samples. High throughput analytical screening technologies are important to quickly identify critical process parameters and to monitor critical product quality attributes. Both protein A and F peptide could easily distinguish samples with low levels of oxidation whereas protein L was not a good ligand for detecting oxidation. Peptide mapping analysis combined with relative binding affinity by SPR analysis allowed for correlations to be made between increases in oxidation at specific methionine residues and decreases in binding to a specific molecular probe. The SPR assay in this study serves as an interesting approach to rapid analysis of oxidation at multiple sites that uses very little material and can be run without any sample prep. One could envision an SPR readout that can accurately estimate the % oxidation at a variety of specific sites if the right molecular probes were used. In addition, other targeted assays could be developed using different molecular probes that are able to detect other stress induced modifications such as deamidation or aggregation. This sort of label-free binding assay could provide rapid results about the integrity of antibody samples and detect a variety of higher order structural changes.

In addition to usefulness in assessing attribute criticality, such selectively oxidized samples also serve as useful and interesting materials for various analytical challenges. Analytical challenge materials are a vital part of demonstrating and determining method capability. Traditional accelerated degradation studies can indeed produce useful materials that can contain a variety of induced amino acid modifications that can challenge various analytical methods. In some cases, the ability of a method to detect and to quantitate a site specific modification might be required, so a more selectively modified challenge material would be of great advantage. These materials with selective modifications can serve as important resources to determine if a method can distinguish site specific modifications versus global changes. For example, the ability of the different LC-MS methods used to characterize oxidized materials in this study is clearly distinguished when comparing IdeS subunit monitoring versus peptide mapping. IdeS subunits can monitor oxidation levels of each subunit (i.e. scFc, Fd, and LC), but peptide mapping is able to determine oxidation at the peptide levels giving us residue specific information. A similar phenomenon can be seen when comparing the SPR and thermal unfolding analysis as the SPR method can give us information about the oxidation levels at specific epitopes while the thermal unfolding studies provide a correlation between oxidation and domain stability. The selectively oxidized CPA samples provided useful and interesting challenge materials for these assays and helped push the limits of each method to detect domain and residue specific oxidation.

## Conclusion

In summary, we developed a strategy to generate uniquely stressed antibody materials by performing the stress in the presence of a bound ligand protein A. These materials were characterized with mass spectrometry to quantitate site specific methionine oxidation. Substantial changes in the oxidation rate and level of multiple methionine residues were shown when compared to materials stressed in solution without bound protein A. With these uniquely oxidized materials in hand, we developed a rapid SPR assay that could detect methionine oxidation in both the Fab and Fc regions using specific molecular probes. The addition of our uniquely oxidized materials to our data set allowed us to hone in on specific methionine residues vital to binding. Further analysis showed that antibody oxidation could also be rapidly detected using thermal unfolding analysis as the stability decreases in multiple domains. The industry relevant stress of accelerated oxidation was used, but other industry relevant stress conditions could be chosen and a similar ligand masking approach could protect specific residues from modification. In the future more studies may reveal other proteins or peptides that can provide site specific protection and use these masking agents to elucidate specific structure function attributes related to a variety of antibody modifications. In addition to monoclonal antibodies, the use of other modalities such as other protein drugs or viral vectors could be of great interest especially when knowledge of structure function relationships is not as developed as mAbs.

## Data Availability

The raw data supporting the conclusion of this article will be made available by the authors, without undue reservation.

## References

[B1] Bertolotti-Ciarlet A. Wang W. Lownes R. Pristatsky P. Fang Y. McKelvey T. (2009). Impact of Methionine Oxidation on the Binding of Human IgG1 to FcRn and Fcγ Receptors. Mol. Immunol. 46, 1878–1882. 10.1016/j.molimm.2009.02.002 19269032

[B2] Chen Y. Doud E. Stone T. Xin L. Hong W. Li Y. (2019). Rapid Global Characterization of Immunoglobulin G1 Following Oxidative Stress. MAbs 11, 1089–1100. 10.1080/19420862.2019.1625676 31156028 PMC6748588

[B3] Chumsae C. Gaza-Bulseco G. Sun J. Liu H. (2007). Comparison of Methionine Oxidation in thermal Stability and Chemically Stressed Samples of a Fully Human Monoclonal Antibody. J. Chromatogr. B 850, 285–294. 10.1016/j.jchromb.2006.11.050 17182291

[B4] Cymer F. Thomann M. Wegele H. Avenal C. Schlothauer T. Gygax D. (2017). Oxidation of M252 but Not M428 in Hu-IgG1 Is Responsible for Decreased Binding to and Activation of Hu-FcγRIIa (His131). Biologicals 50, 125–128. 10.1016/j.biologicals.2017.09.006 28988621

[B5] Dashivets T. Stracke J. Dengl S. Knaupp A. Pollmann J. Buchner J. (2016). Oxidation in the Complementarity-Determining Regions Differentially Influences the Properties of Therapeutic Antibodies. mAbs 8, 1525–1535. Taylor & Francis. 10.1080/19420862.2016.1231277 27612038 PMC5098445

[B6] Deis L. N. Wu Q. Wang Y. Qi Y. Daniels K. G. Zhou P. (2015). Suppression of Conformational Heterogeneity at a Protein-Protein Interface. Proc. Natl. Acad. Sci. U.S.A. 112, 9028–9033. 10.1073/pnas.1424724112 26157136 PMC4517213

[B7] Gallagher D. T. Galvin C. V. Karageorgos I. (2018). Structure of the Fc Fragment of the NIST Reference Antibody RM8671. Acta Cryst. Sect F 74, 524–529. 10.1107/s2053230x18009834 PMC6130425 30198883

[B8] Gao X. Ji J. A. Veeravalli K. John Wang Y. Zhang T. Mcgreevy W. (2015). Effect of Individual Fc Methionine Oxidation on FcRn Binding: Met252 Oxidation Impairs FcRn Binding More Profoundly Than Met428 Oxidation. J. Pharm. Sci. 104, 368–377. 10.1002/jps.24136 25175600

[B9] GE Healthcare (2004). MabSelect SuRe-studies on Ligand Toxicity, Leakage, Removal of Leached Ligand, and Sanitization; 11-0011-64 AA. Amersham: Amersham Application Note 2004, Process-scale antibody purification.

[B10] Gokarn Y. Agarwal S. Arthur K. Bepperling A. Day E. S. Filoti D. (2015). “Biophysical Techniques for Characterizing the Higher Order Structure and Interactions of Monoclonal Antibodies,” in State-of-the-Art and Emerging Technologies for Therapeutic Monoclonal Antibody Characterization Volume 2. Biopharmaceutical Characterization: The NISTmAb Case Study (ACS Publications), 285–327. 10.1021/bk-2015-1201.ch006

[B11] Haberger M. Bomans K. Diepold K. Hook M. Gassner J. Schlothauer T. (2014). Assessment of Chemical Modifications of Sites in the CDRs of Recombinant Antibodies: Susceptibility vs. Functionality of Critical Quality Attributes. MAbs 6, 327–339. 10.4161/mabs.27876 24441081 PMC3984323

[B12] Jansson B. Uhlén M. Nygren P.-Å. (1998). All Individual Domains of Staphylococcal Protein A Show Fab Binding. FEMS Immunol. Med. Microbiol. 20, 69–78. 10.1016/s0928-8244(97)00108-9 9514577

[B13] Karlsson R. Fridh V. Frostell Å. (2018). Surrogate Potency Assays: Comparison of Binding Profiles Complements Dose Response Curves for Unambiguous Assessment of Relative Potencies. J. Pharm. Anal. 8, 138–146. 10.1016/j.jpha.2017.12.008 29736301 PMC5934736

[B14] Li W. Kerwin J. L. Schiel J. Formolo T. Davis D. Mahan A. (2015). “Structural Elucidation of post-translational Modifications in Monoclonal Antibodies,” in State-of-the-art and Emerging Technologies for Therapeutic Monoclonal Antibody Characterization Volume 2. Biopharmaceutical Characterization: The NISTmAb Case Study (ACS Publications), 119–183. 10.1021/bk-2015-1201.ch003

[B15] Ljungberg U. K. Jansson B. Niss U. Nilsson R. Sandberg B. E. B. Nilsson B. (1993). The Interaction between Different Domains of Staphylococcal Protein A and Human Polyclonal IgG, IgA, IgM and F(ab')2: Separation of Affinity from Specificity. Mol. Immunol. 30, 1279–1285. 10.1016/0161-5890(93)90044-c 8413328

[B16] Mo J. Yan Q. So C. K. Soden T. Lewis M. J. Hu P. (2016). Understanding the Impact of Methionine Oxidation on the Biological Functions of IgG1 Antibodies Using Hydrogen/deuterium Exchange Mass Spectrometry. Anal. Chem. 88, 9495–9502. 10.1021/acs.analchem.6b01958 27575380

[B17] Mouchahoir T. Schiel J. E. (2018). Development of an LC-MS/MS Peptide Mapping Protocol for the NISTmAb. Anal. Bioanal. Chem. 410, 2111–2126. 10.1007/s00216-018-0848-6 29411091 PMC5830484

[B18] Pan H. Chen K. Chu L. Kinderman F. Apostol I. Huang G. (2009). Methionine Oxidation in Human IgG2 Fc Decreases Binding Affinities to Protein A and FcRn. Protein Sci. 18, 424–433. 10.1002/pro.45 19165723 PMC2708056

[B19] Schiel J. E. Davis D. L. Borisov O. (2015). “State-of-the-art and Emerging Technologies for Therapeutic Monoclonal Antibody Characterization Volume 3. Defining the Next Generation of Analytical and Biophysical Techniques,” in ACS Symposium Series (ACS Publications), 455.

[B20] Sokolowska I. Mo J. Dong J. Lewis M. J. Hu P. (2017). Subunit Mass Analysis for Monitoring Antibody Oxidation. mAbs 9, 498–505. Taylor & Francis. 10.1080/19420862.2017.1279773 28106519 PMC5384710

[B21] Stracke J. Emrich T. Rueger P. Schlothauer T. Kling L. Knaupp A. (2014). A Novel Approach to Investigate the Effect of Methionine Oxidation on Pharmacokinetic Properties of Therapeutic Antibodies. mAbs 6, 1229–1242. Taylor & Francis. 10.4161/mabs.29601 25517308 PMC4622569

[B22] Turner A. Yandrofski K. Telikepalli S. King J. Heckert A. Filliben J. (2018). Development of Orthogonal NISTmAb Size Heterogeneity Control Methods. Anal. Bioanal. Chem. 410, 2095–2110. 10.1007/s00216-017-0819-3 29428991 PMC5830496

[B23] Yang R. Jain T. Lynaugh H. Nobrega R. P. Lu X. Boland T. (2017). Rapid Assessment of Oxidation via Middle-Down LCMS Correlates with Methionine Side-Chain Solvent-Accessible Surface Area for 121 Clinical Stage Monoclonal Antibodies. MAbs 9, 646–653. 10.1080/19420862.2017.1290753 28281887 PMC5419077

