# Supplementary figures and images for: Ligand-Bound Forced Degradation as a Strategy to Generate Functionally Relevant Analytical Challenge Materials for Assessment of CQAs
Source: Front Mol Biosci. 2022 Apr 11;9:789973. doi: 10.3389/fmolb.2022.789973 (PMC9035890; doi:10.3389/fmolb.2022.789973)

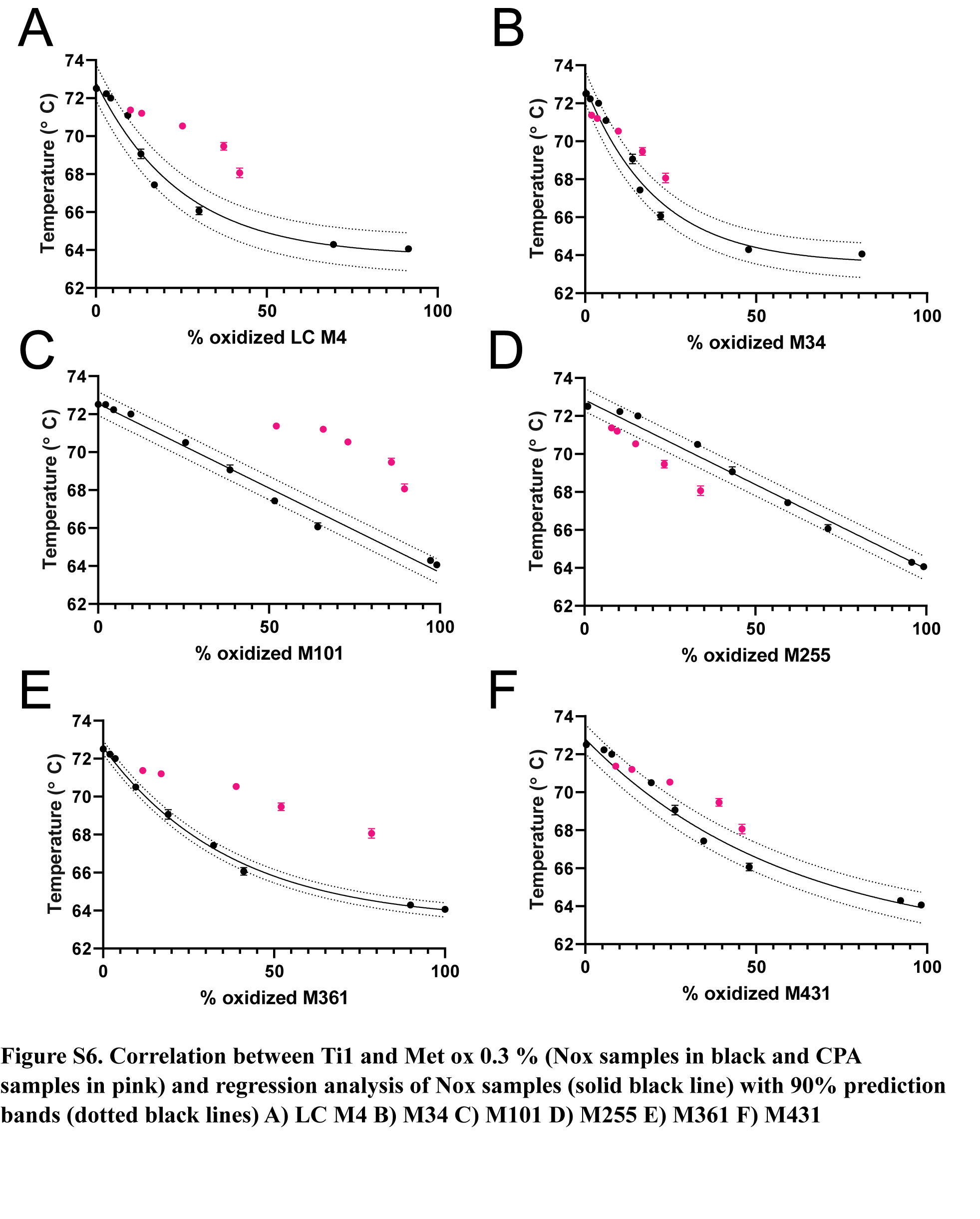

Supplement: Supplementary file 1 [file Image6.TIF]

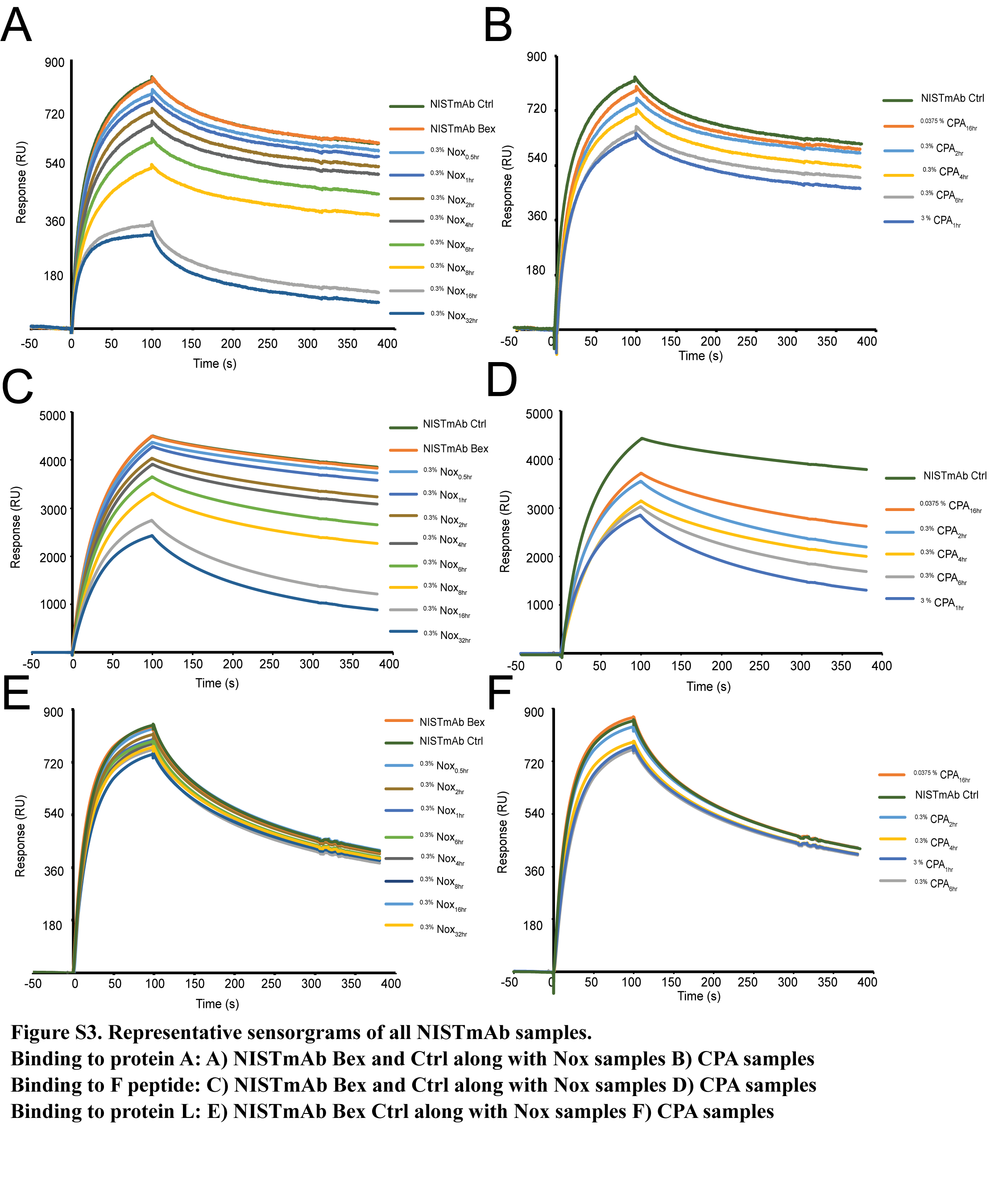

Supplement: Supplementary file 2 [file Image3.TIF]

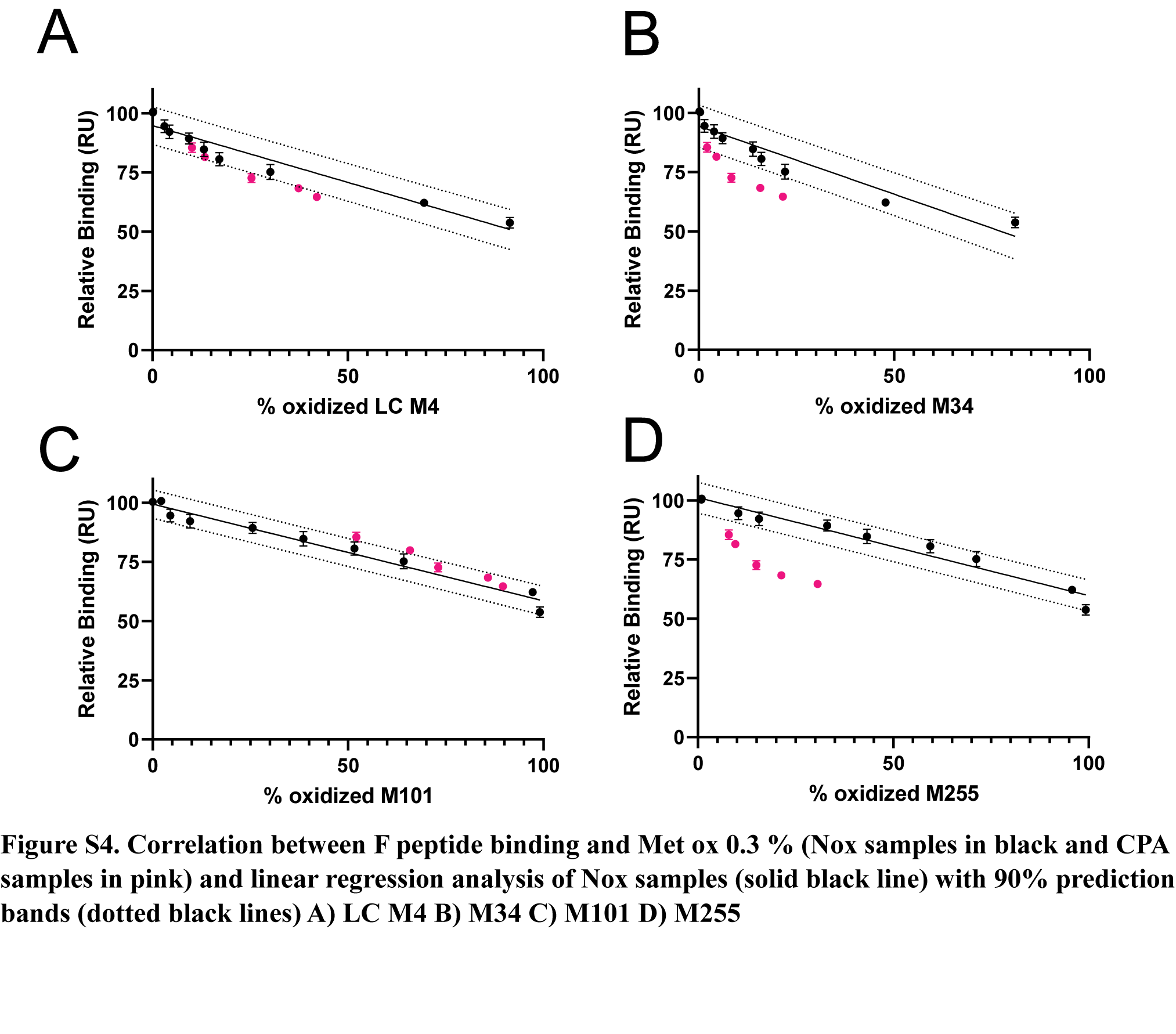

Supplement: Supplementary file 3 [file Image4.TIF]

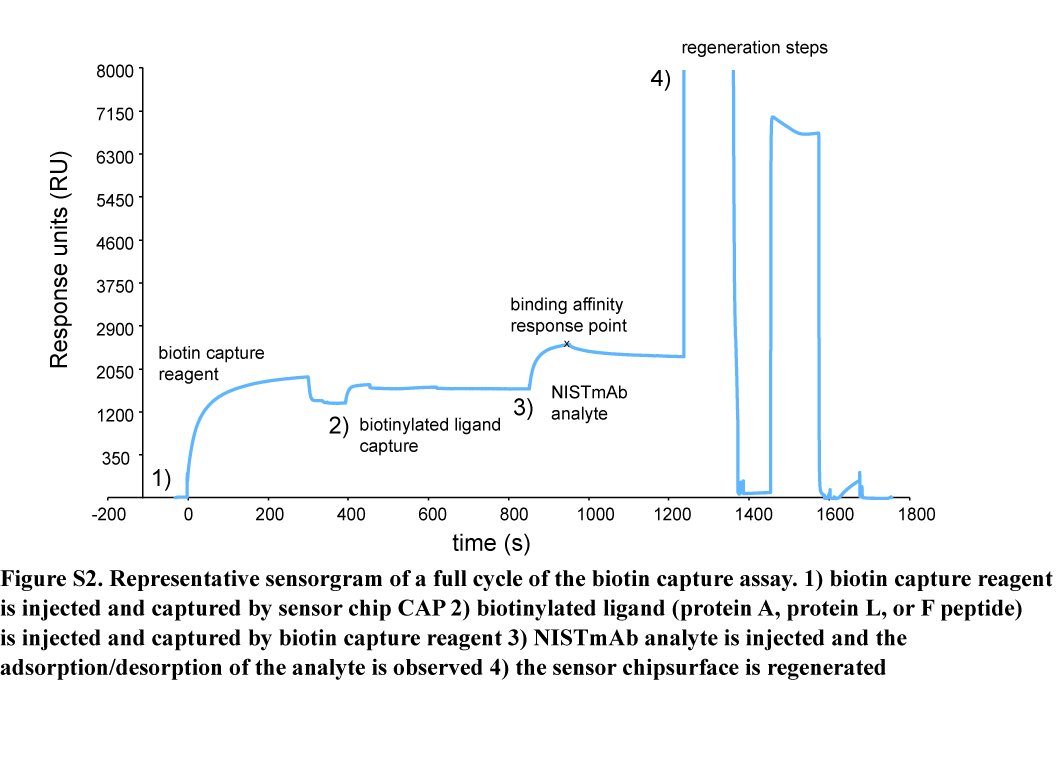

Supplement: Supplementary file 4 [file Image2.tif]

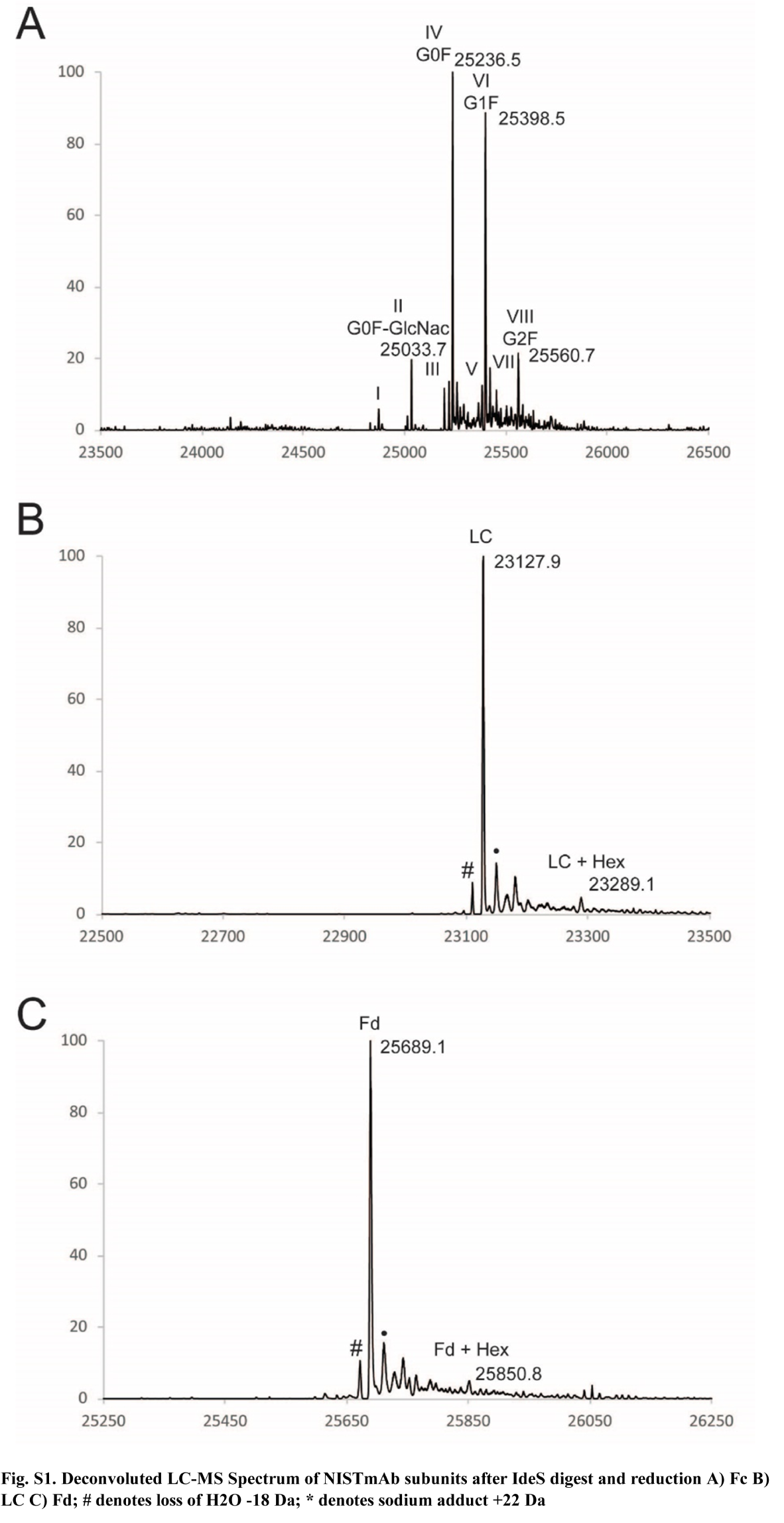

Supplement: Supplementary file 5 [file Image1.TIF]

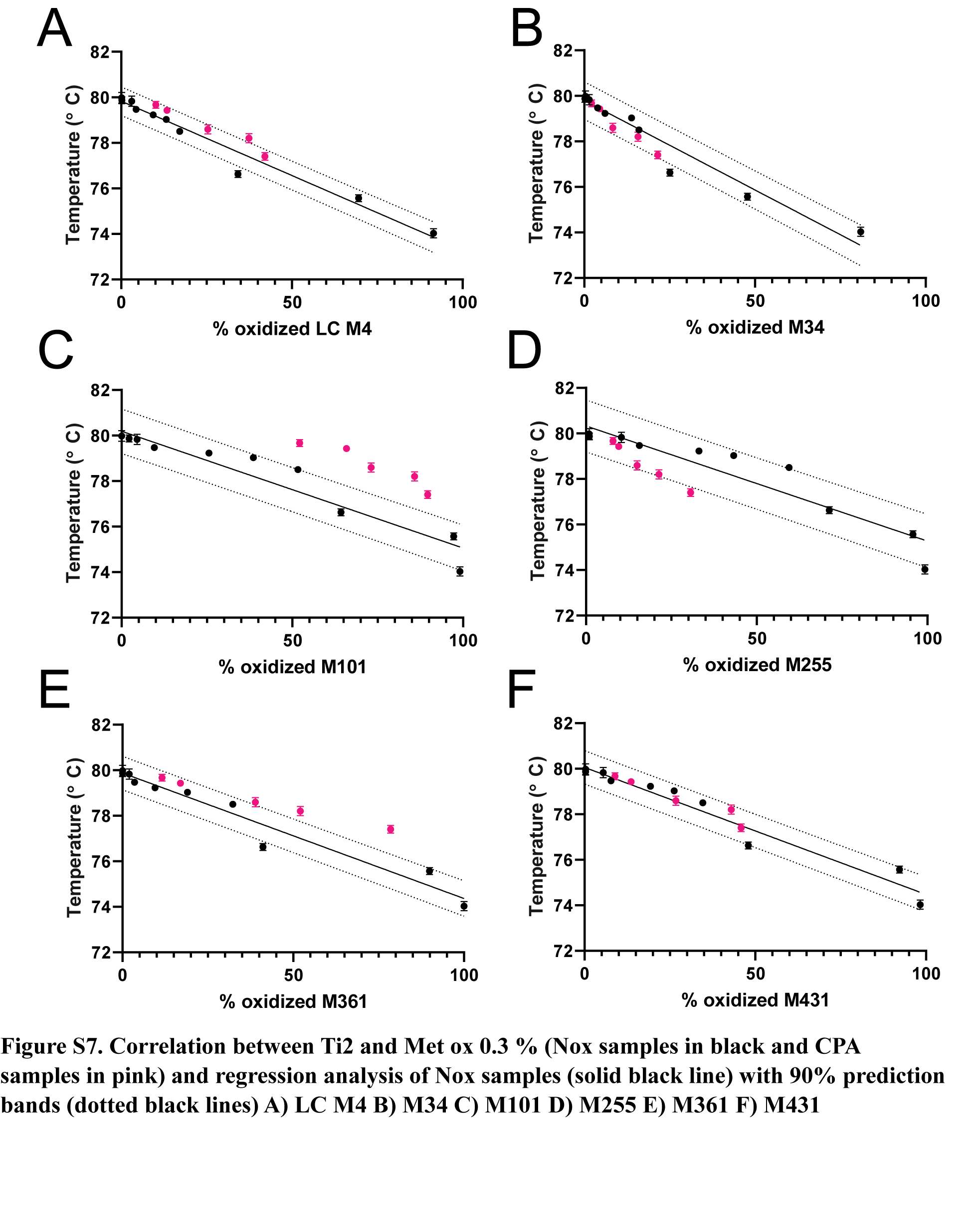

Supplement: Supplementary file 6 [file Image7.TIF]

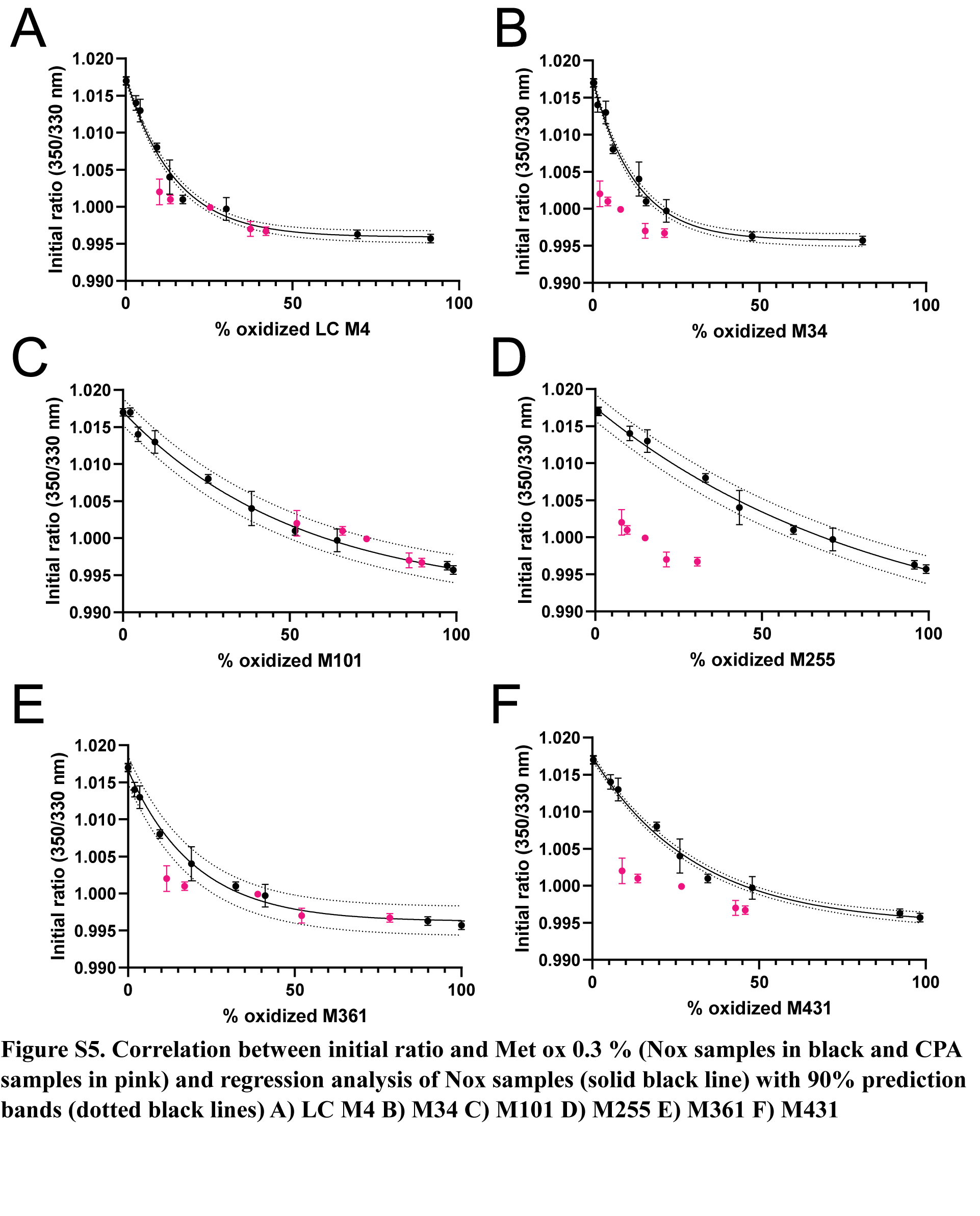

Supplement: Supplementary file 7 [file Image5.TIF]
