# Supplementary material for: Ligand-Bound Forced Degradation as a Strategy to Generate Functionally Relevant Analytical Challenge Materials for Assessment of CQAs
Source: Front Mol Biosci. 2022 Apr 11;9:789973. doi: 10.3389/fmolb.2022.789973 (PMC9035890; doi:10.3389/fmolb.2022.789973)
Supplement: Supplementary file 8 [file DataSheet1.docx]

**Supplemental Tables**

Table S1. Mass assignments for IdeS fragments of NISTmAb

| IdeS Fragment | Peak | Assignment | Obs. Mass (Da) | Calc Mass (Da) | Deviation (ppm) |
| --- | --- | --- | --- | --- | --- |
| **scFc** |  |  |  |  |  |
|  | I | H2N3F1 | 24871.4 | 24870.8 | 25.4 |
|  | II | H3N3F1 | 25033.6 | 25032.9 | 27.6 |
|  | III | H4N3F1 | 25195.6 | 25195.0 | 23.7 |
|  | IV | H3N4F1 (G0F) | 25236.5 | 25236.1 | 15.7 |
|  | V | H3N4F1 (G0F) + Lys | 25363.9 | 25364.3 | -14.9 |
|  | VI | H4N4F1 (G1F) | 25398.5 | 25398.2 | 10.0 |
|  | VII | H4N4F1 (G1F) + Lys | 25527.3 | 25526.4 | 34.6 |
|  | VIII | H5N4F1 (G2F) | 25560.7 | 25560.4 | 12.3 |
| **LC** |  |  |  |  |  |
|  |  | LC | 23127.9 | 23127.6 | 11.9 |
|  |  | LC+Hex | 23289.1 | 23289.8 | -28.6 |
| **Fd** |  |  |  |  |  |
|  |  | Fd | 25689.1 | 25689.0 | 4.2 |
|  |  | Fd+Hex | 25850.8 | 25851.1 | -12.8 |

|  | |  | |  | | |  | |  |
| --- | --- | --- | --- | --- | --- | --- | --- | --- | --- |
|  |  | |  | |  |  | |  | |

Table S2. Ligand capture level (Intra Assay) n=20

|  | Protein A | F peptide | Protein L |
| --- | --- | --- | --- |
| Mean (RU) | 306.1 | 304.7 | 224.8 |
| St Dev | 10.1 | 3.1 | 6.9 |
| % CV | 3.3 | 1.0 | 3.1 |

Table S3. NISTmAb Bex binding level (Intra Assay) n=20

|  | Protein A | F peptide | Protein L |
| --- | --- | --- | --- |
| Mean (RU) | 793.6 | 4379.5 | 820.6 |
| St Dev | 25.4 | 34.3 | 25.3 |
| % CV | 3.2 | 0.8 | 3.1 |

Table S4. Protein A capture level

|  | Exp 1 | Exp 2 | Exp 3 | Inter Assay |
| --- | --- | --- | --- | --- |
| Mean (RU) | 277.8 | 293.9 | 306.1 | 292.6 |
| St Dev | 4.93 | 5.29 | 2.60 | 14.19 |
| % CV | 1.8 | 1.8 | 0.9 | 4.9 |

Table S5. F peptide capture level

|  | Exp 1 | Exp 2 | Exp 3 | Inter Assay |
| --- | --- | --- | --- | --- |
| Mean (RU) | 306.4 | 300.9 | 297.2 | 301.5 |
| St Dev | 1.23 | 1.30 | 0.94 | 4.63 |
| % CV | 0.4 | 0.4 | 0.3 | 1.5 |

Table S6. Protein L capture level

|  | Exp 1 | Exp 2 | Exp 3 | Inter Assay |
| --- | --- | --- | --- | --- |
| Mean (RU) | 231.8 | 226.0 | 230.4 | 229.4 |
| St Dev | 4.43 | 1.31 | 1.01 | 3.02 |
| % CV | 1.9 | 0.6 | 0.4 | 1.3 |

Table S7. SEC analysis

|  | HMW | Monomer | LMW |
| --- | --- | --- | --- |
| NISTmAb Bex | 2.344 | 97.479 | 0.177 |
| NISTmAb Ctrl | 1.702 | 98.121 | 0.177 |
| ^0.3 %^Nox_0.5hr_ | 2.244 | 97.584 | 0.173 |
| ^0.3 %^Nox_1hr_ | 2.134 | 97.69 | 0.176 |
| ^0.3 %^Nox_2hr_ | 2.138 | 97.686 | 0.175 |
| ^0.3 %^Nox_4hr_ | 2.088 | 97.732 | 0.18 |
| ^0.3 %^Nox_6hr_ | 2.228 | 97.587 | 0.185 |
| ^0.3 %^Nox_8hr_ | 1.906 | 97.904 | 0.19 |
| ^0.3 %^Nox_16hr_ | 1.83 | 97.962 | 0.208 |
| ^0.3 %^Nox_32hr_ | 1.487 | 98.275 | 0.238 |
| ^0.0375 %^CPA_16hr_ | 5.61 | 94.078 | 0.313 |
| ^0.3 %^CPA_2hr_ | 2.709 | 97.158 | 0 |
| ^0.3 %^CPA_4hr_ | 2.842 | 97.158 | 0 |
| ^0.3 %^CPA_6hr_ | 2.577 | 97.423 | 0 |
| ^3 %^CPA_1hr_ | 2.775 | 97.225 | 0 |

Table S8: Standard error of regression (Sy.x) of Nox and CPA data points for protein A relative binding vs % Met oxidized

| Methionine | Nox | CPA |
| --- | --- | --- |
| M101 | 4.3 | 33.4 |
| M255 | 5.6 | 5.1 |
| M361 | 2.8 | 21.8 |
| M431 | 2.0 | 10.3 |

Table S9: Standard error of regression (Sy.x) of Nox and CPA data points for F peptide relative binding vs % Met oxidized

| Methionine | Nox | CPA |
| --- | --- | --- |
| LC M4 | 4.5 | 8.9 |
| M34 | 5.1 | 15.6 |
| M101 | 3.3 | 5.0 |
| M255 | 3.7 | 21.8 |

Table S10. P-values SPR data

|  | Protein A | F peptide |
| --- | --- | --- |
| NISTmAb Ctrl | 0.7164 | 0.445 |
| ^0.3 %^Nox_0.5hr_ | 0.0028 | 0.0008 |
| ^0.3 %^Nox_1hr_ | 0.0001 | 0.0001 |
| ^0.3 %^Nox_2hr_ | 0.0001 | 0.0001 |
| ^0.3 %^Nox_4hr_ | 0.0001 | 0.0001 |
| ^0.3 %^Nox_6hr_ | 0.0001 | 0.0001 |
| ^0.3 %^Nox_8hr_ | 0.0001 | 0.0001 |
| ^0.3 %^Nox_16hr_ | 0.0001 | 0.0001 |
| ^0.3 %^Nox_32hr_ | 0.0001 | 0.0001 |

Table S11: Standard error of regression (Sy.x) of Nox and CPA data points for initial ratio vs % Met oxidized

| Methionine | Nox | CPA |
| --- | --- | --- |
| LC M4 | 1.2 ×10^-3^ | 5.6 ×10^-3^ |
| M34 | 1.2 ×10^-3^ | 17.5 ×10^-3^ |
| M101 | 1.0 ×10^-3^ | 2.4 ×10^-3^ |
| M255 | 1.0 ×10^-3^ | 27.3 ×10^-3^ |
| M361 | 1.1 ×10^-3^ | 7.2 ×10^-3^ |
| M431 | 1.1 ×10^-3^ | 13.9 ×10^-3^ |

Table S12: Standard error of regression (Sy.x) of Nox and CPA data points for Ti_1_ vs % Met oxidized

| Methionine | Nox | CPA |
| --- | --- | --- |
| LC M4 | 0.54 | 6.4 |
| M34 | 0.49 | 2.6 |
| M101 | 0.36 | 5.0 |
| M255 | 0.35 | 1.7 |
| M361 | 0.20 | 6.9 |
| M431 | 0.44 | 2.8 |

Table S13: Standard error of regression (Sy.x) of Nox and CPA data points for Ti_2_ vs % Met oxidized

| Methionine | Nox | CPA |
| --- | --- | --- |
| LC M4 | 0.36 | 0.69 |
| M34 | 0.48 | 0.56 |
| M101 | 0.54 | 2.9 |
| M255 | 0.60 | 1.1 |
| M361 | 0.43 | 1.4 |
| M431 | 0.42 | 0.33 |
